# Supplementary material for: Micropattern differentiation of mouse pluripotent stem cells recapitulates embryo regionalized cell fate patterning
Source: eLife. 2018 Feb 7;7:e32839. doi: 10.7554/eLife.32839 (PMC5807051; doi:10.7554/eLife.32839)

Additional Data Resource

NANOG_SOX2_BRACHYURY
i. E6.5-E6.75: Wholemount imaging
ii. E6.5-E6.75: Cryosections
iii. E7.0-E7.25: Wholemount imaging NANOG_SOX2
iv. E7.5: Wholemount imaging
v. E7.5: Cryosections
vi. E8.0: Wholemount imaging
vii. E8.0: Cryosections

CDX2_BRACHYURY_GATA6
viii. E6.5-E6.75: Wholemount imaging/cryosections
ix. E7.0-E7.25: Wholemount imaging
x. E7.0-E7.25: Cryosections
xi. E7.5: Wholemount imaging
xii. E7.75: Wholemount imaging
xiii. E7.75: Cryosections

GATA6_CDX2_SOX17
xiv. E6.5-E6.75: Wholemount imaging
xv. E6.5-E6.75: Cryosections
xvi. E7.75-E8.0: Wholemount imaging
xvii. E7.75-E8.0: Cryosections and E8.5 allantois cryosection

FOXA2_CDX2_BRACHYURY
xviii. E7.0-E7.25: Wholemount imaging/cryosections
xix. E7.5-E7.75: Wholemount imaging
xx. E7.5-E7.75: Cryosections

FOXA2_SOX17_BRACHYURY
xxi. E7.0-E7.25: Wholemount imaging/cryosections
xxii. E7.75-E8.0: Wholemount imaging
xxiii. E7.75-E8.0: Cryosections

FOXF1_GATA6_CDX2
xxiv. E6.5-E6.75 and E7.75-E8.0: Wholemount imaging
xxv. E7.75-E8.0: Cryosections

NANOG_SOX2_OTX2
xxvi. E6.25 (pre-streak) and E7.5: Wholemount imaging
xxvii. E7.5: Cryosections
xxviii. E7.75: Wholemount imaging/cryosections

pSMAD1/5/8_BRACHYURY_CDH1
xxix. E6.5-E6.75: Wholemount imaging/cryosections
xxx. E7.0-E7.25: Wholemount imaging
xxxi. E7.75-E8.0: Wholemount imaging/cryosections

ABBREVIATIONS:
MIP, maximum intensity projection
A, anterior
P, posterior
Pr, proximal
D, distal
L, left
R, right

Al, allantois
Am, amnion
AxM, axial mesoderm
CM, cardiac mesoderm
E, embryonic day
Epi, epiblast
En, endoderm
ExE, extraembryonic ectoderm
ExM, extraembryonic mesoderm
Fg, foregut
HF, headfold
HM, head mesenchyme
M1, embryonic mesoderm 1
M2, embryonic mesoderm 2
Meso, embryonic mesoderm
ML, midline
NE, neurectoderm
P-Epi, posterior epiblast
VE, visceral endoderm
YSE, yolk sac endoderm
YSM, yolk sac mesoderm

Scale bars, 50 μm unless otherwise stated. Bracket demarcates the primitive streak. Dashed lines indicate approximate plane of transverse confocal optical section. Dashed boxes demarcate regions shown in higher magnification.

Morgani et al., i. NANOG, BRACHYURY, SOX2: E6.5-E6.75

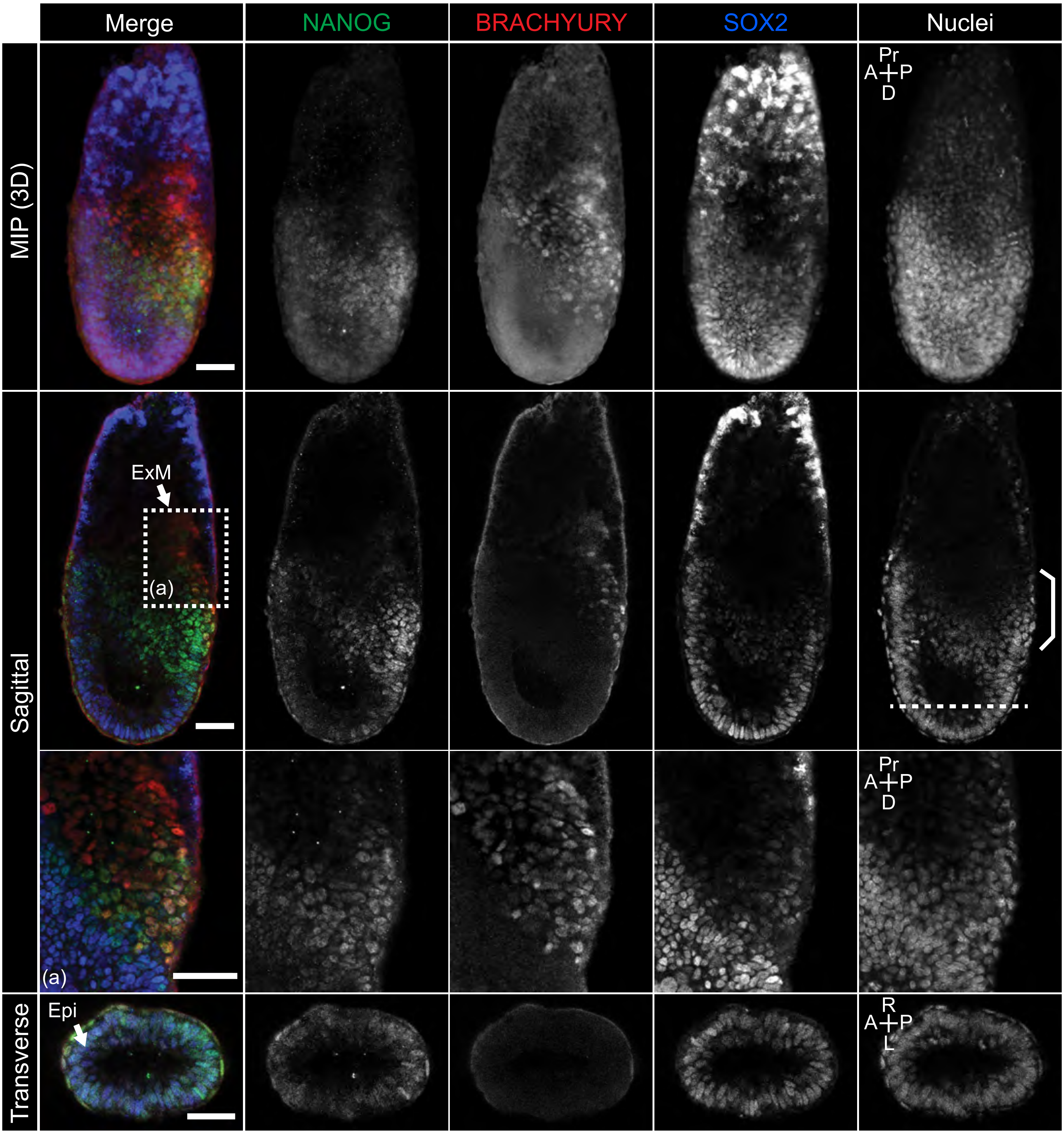

Morgani et al., iii. NANOG, BRACHYURY, SOX2: E6.5-E6.75

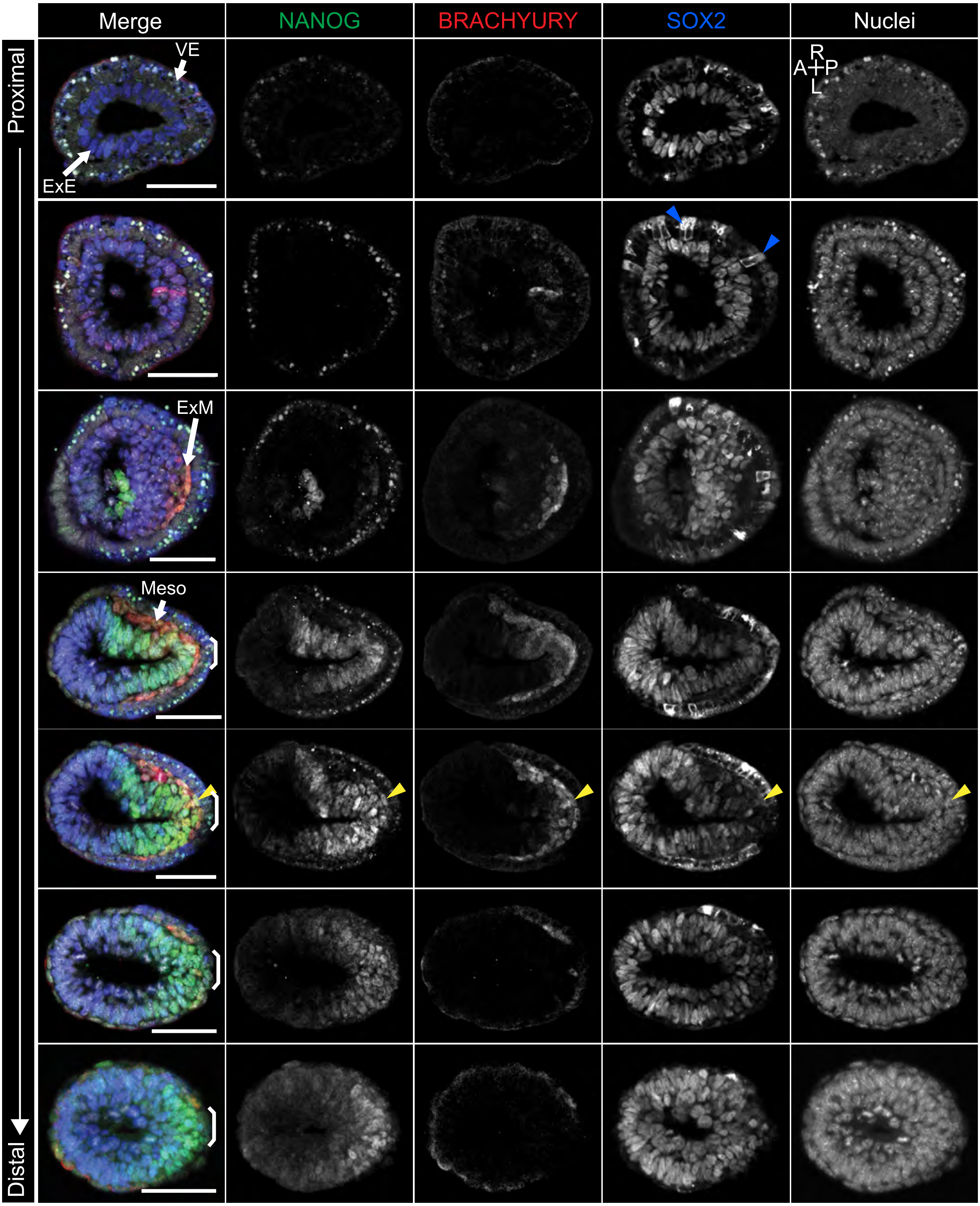

Blue arrowhead: Non-nuclear SOX2 staining in extraembryonic visceral endoderm.
Yellow arrowhead: NANOG/SOX2/BRACHYURY -expressing cell.

Morgani et al., iii. NANOG, SOX2: E7.0-E7.25

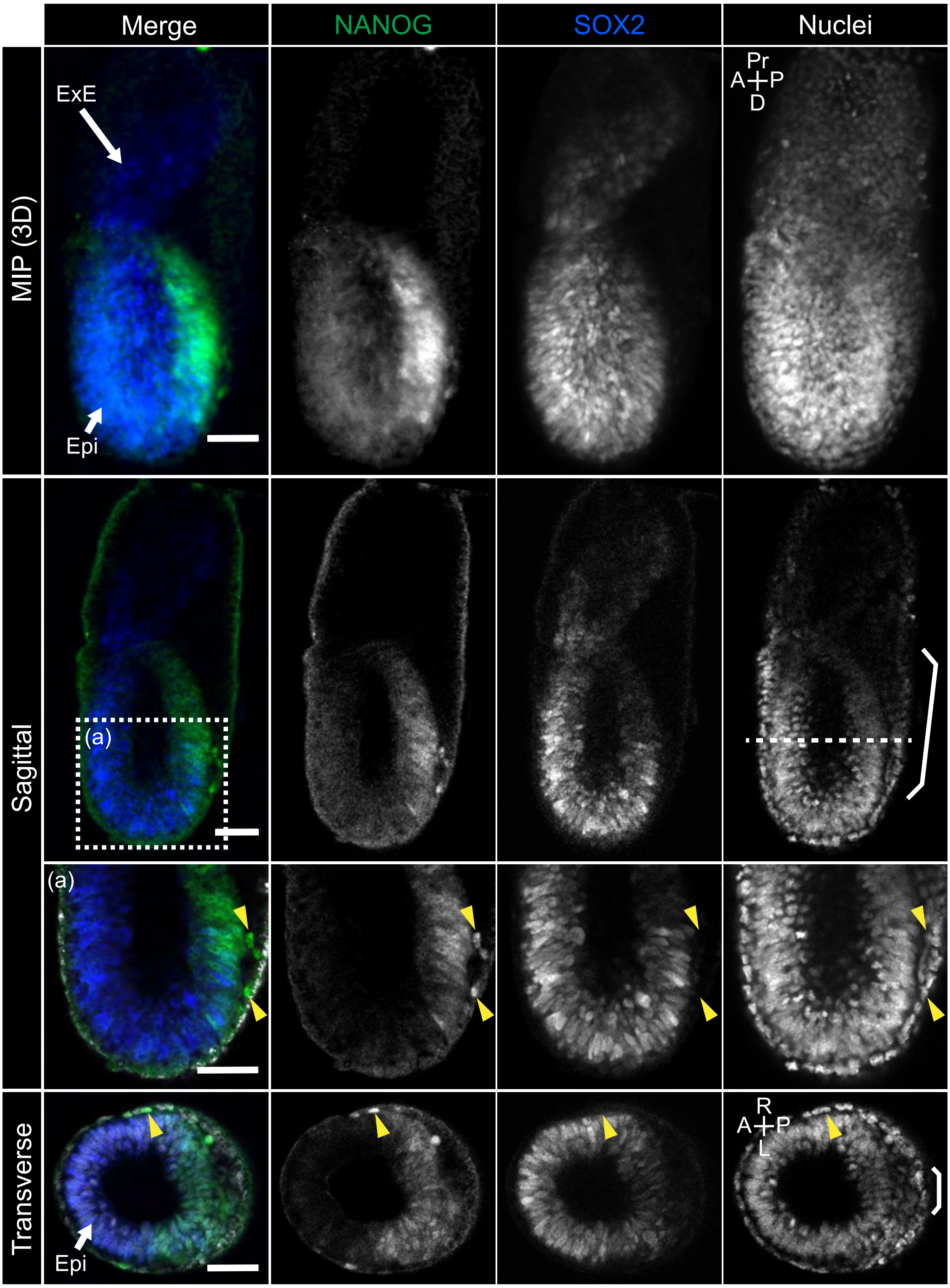

Yellow arrowhead: NANOG-expressing cells within anterior primitive streak and endoderm.

Morgani et al., iv. NANOG, BRACHYURY, SOX2: E7.5

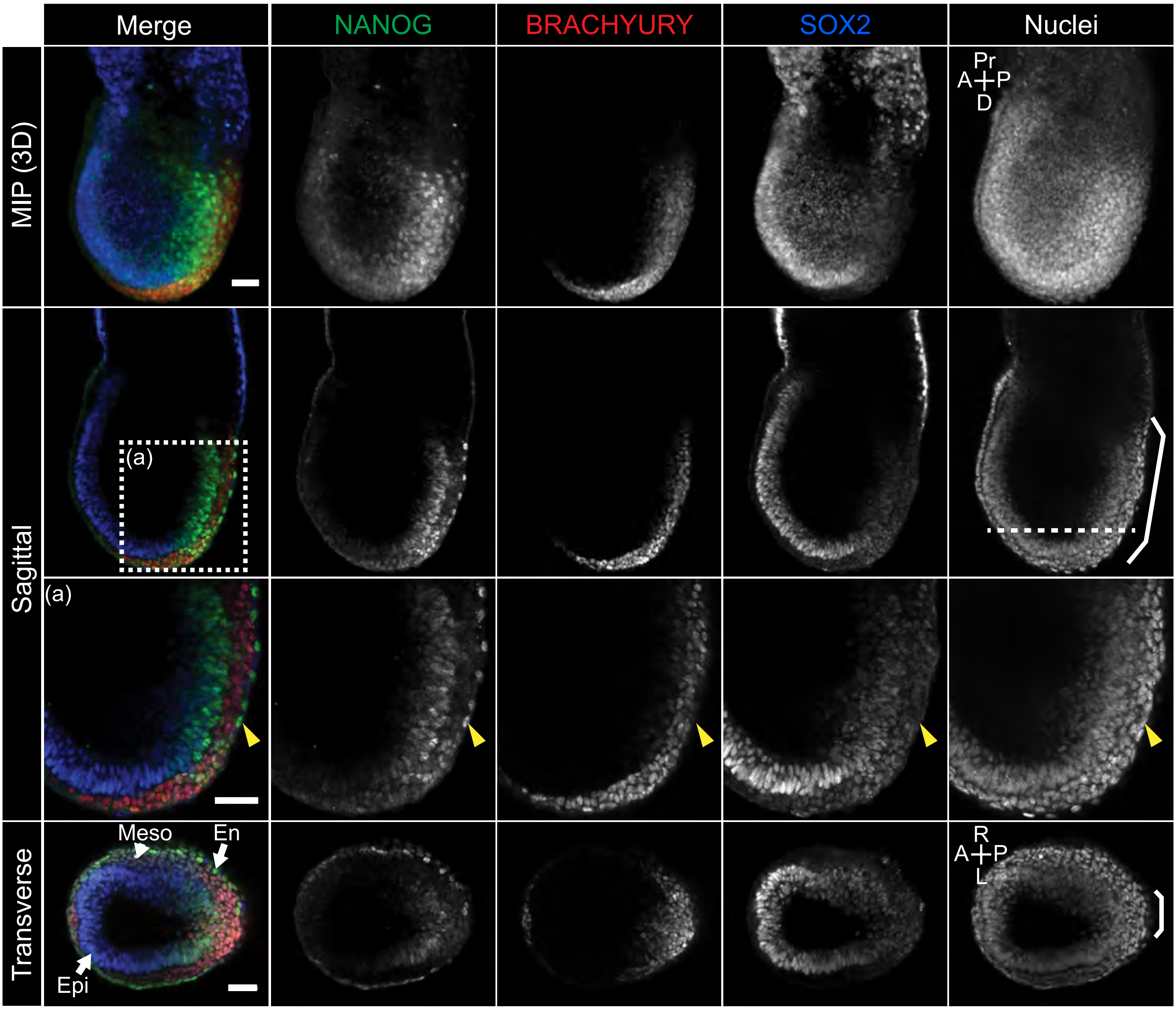

Yellow arrowhead: NANOG-expressing cells within the outer endoderm layer.

Morgani et al., v. NANOG, BRACHYURY, SOX2: E7.5

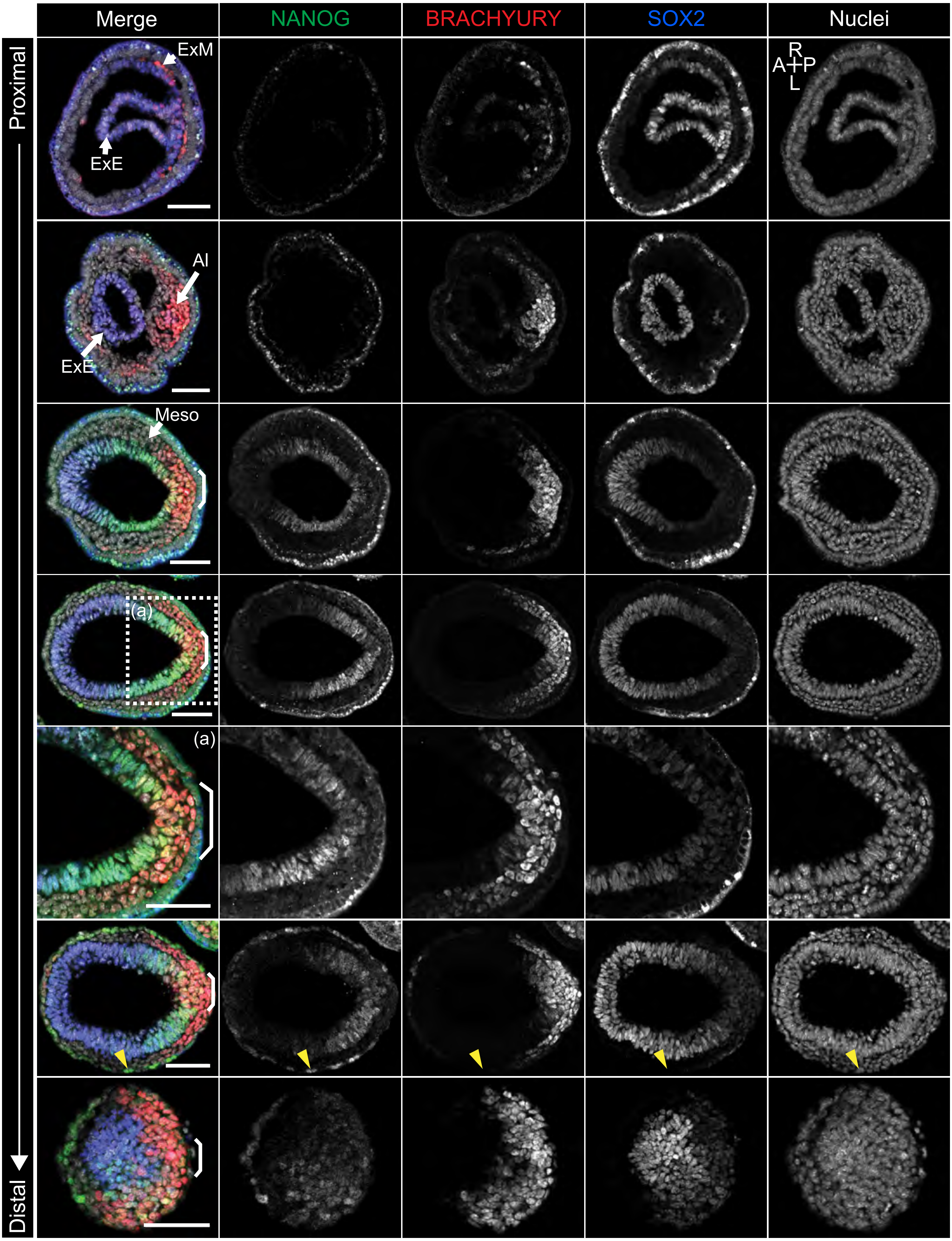

Yellow arrowhead: NANOG-expressing cells within the outer endoderm layer.

Morgani et al., vi. NANOG, BRACHYURY, SOX2: E8.0

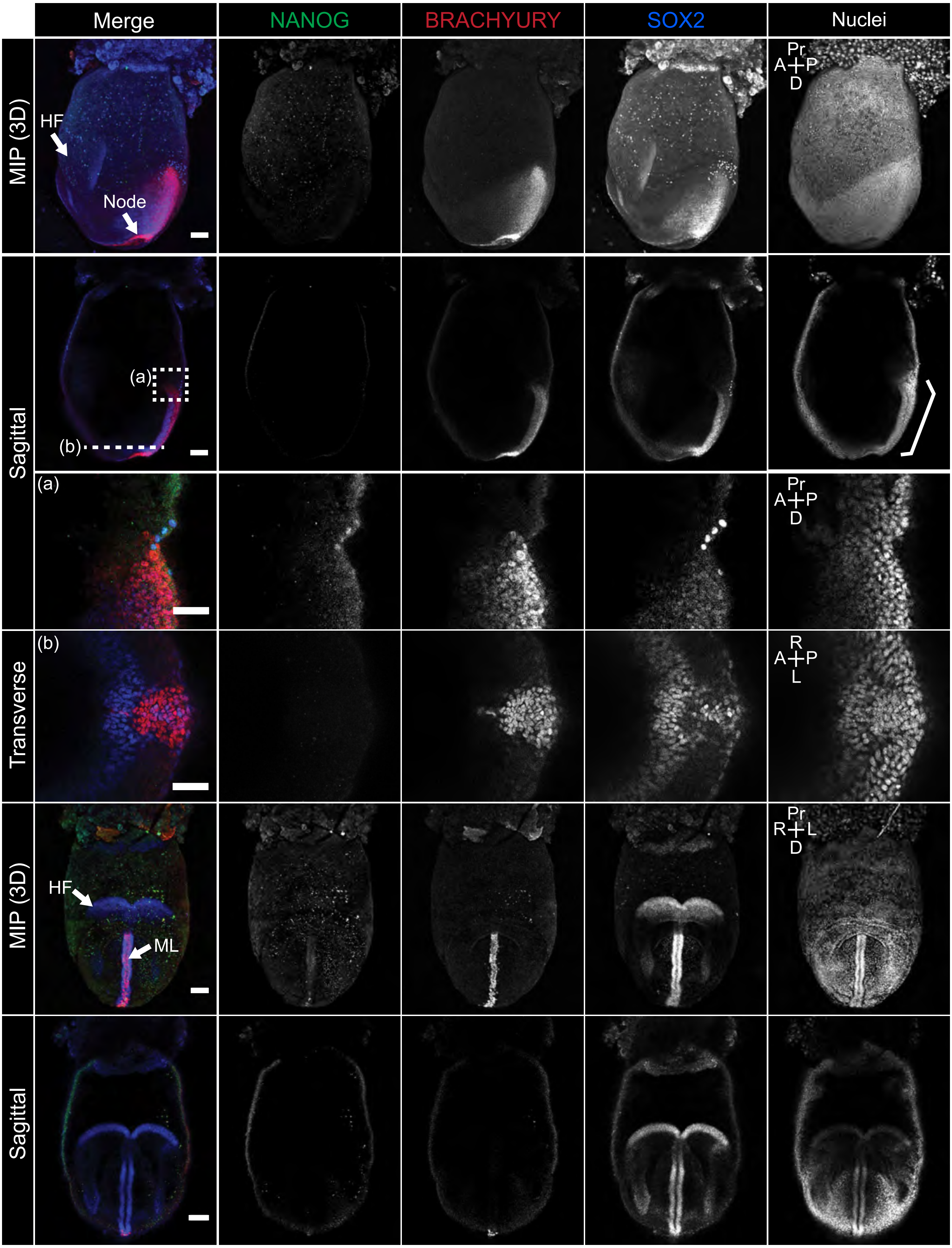

Scale bars, 100 μm and 50 μm high magnification images depicted in panels (a) and (b).

Morgani et al., vii. NANOG, BRACHYURY, SOX2: E8.0

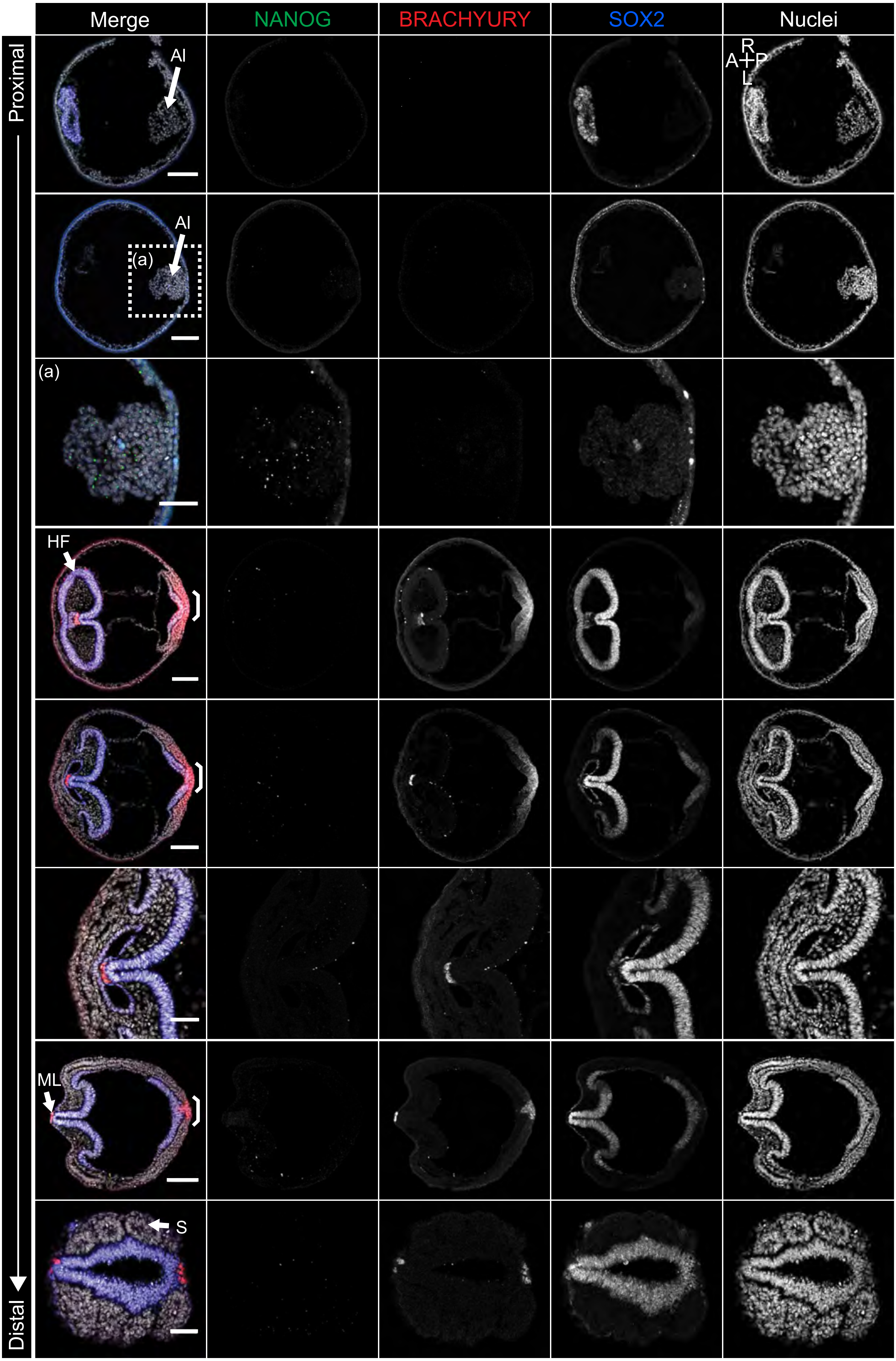

Morgani et al., viii. CDX2, BRACHYURY, GATA6: E6.5-E6.75

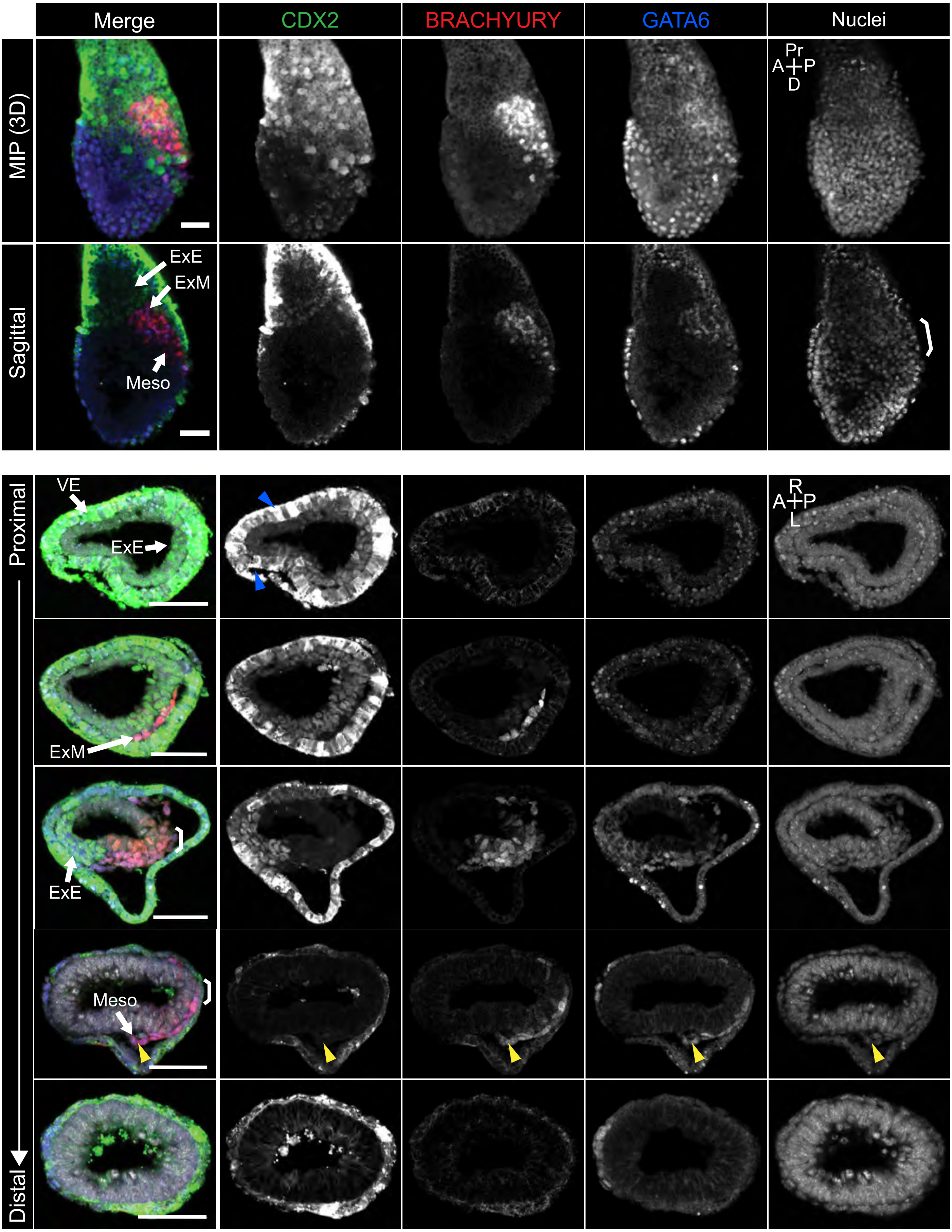

Yellow arrowhead indicates BRACHYURY/GATA6 -expressing cell.
Blue arrowhead: Non-nuclear CDX2 staining in extraembryonic visceral endoderm.

Morgani et al., ix. CDX2, BRACHYURY, GATA6: E7.0-E7.25

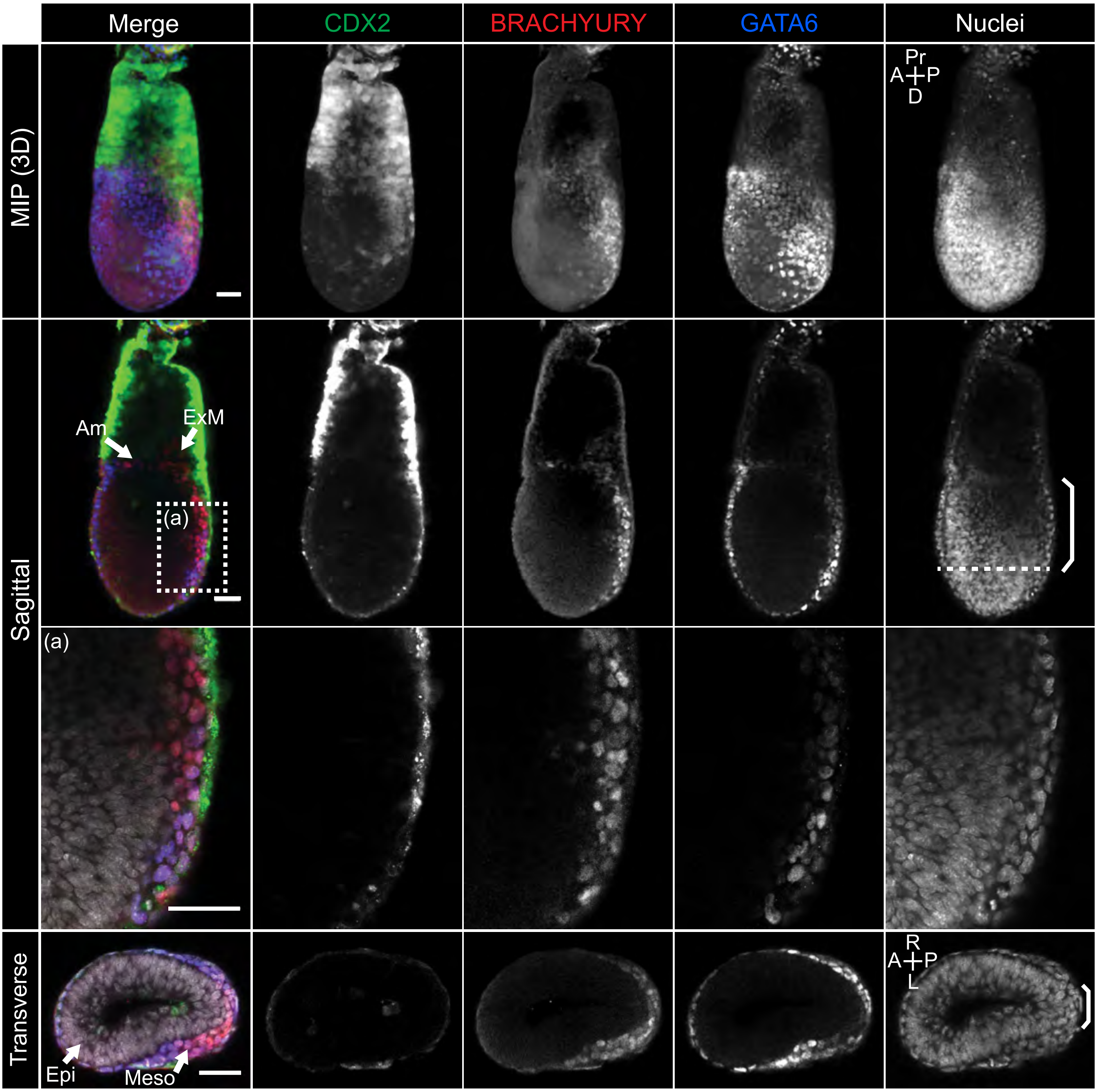

Morgani et al., x. CDX2, BRACHYURY, GATA6: E7.0-E7.25

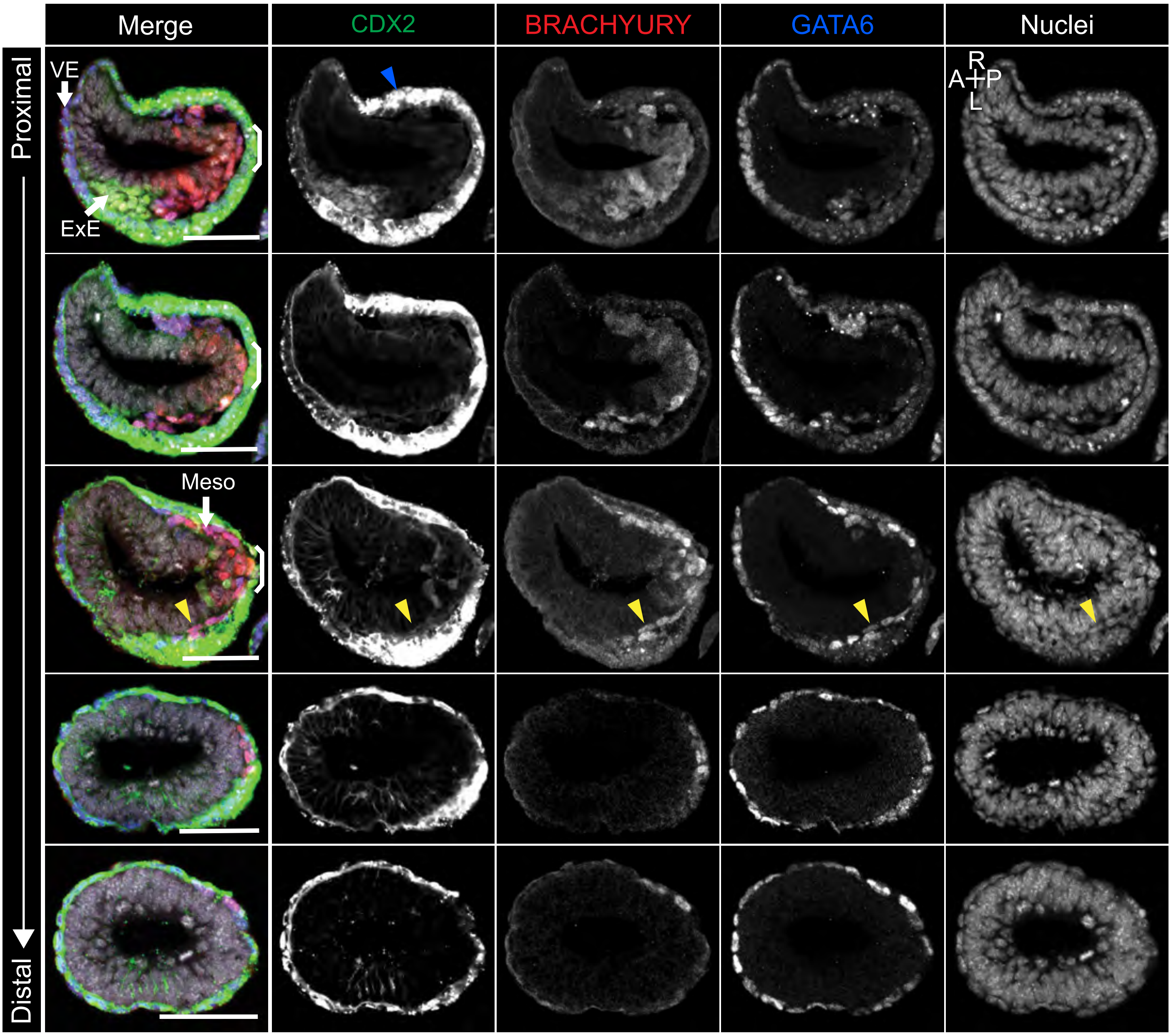

Yellow arrowhead indicates BRACHYURY/GATA6 -expressing cell.
Blue arrowhead: Non-nuclear CDX2 staining in visceral endoderm.

Morgani et al., xi. CDX2, BRACHYURY, GATA6: E7.5

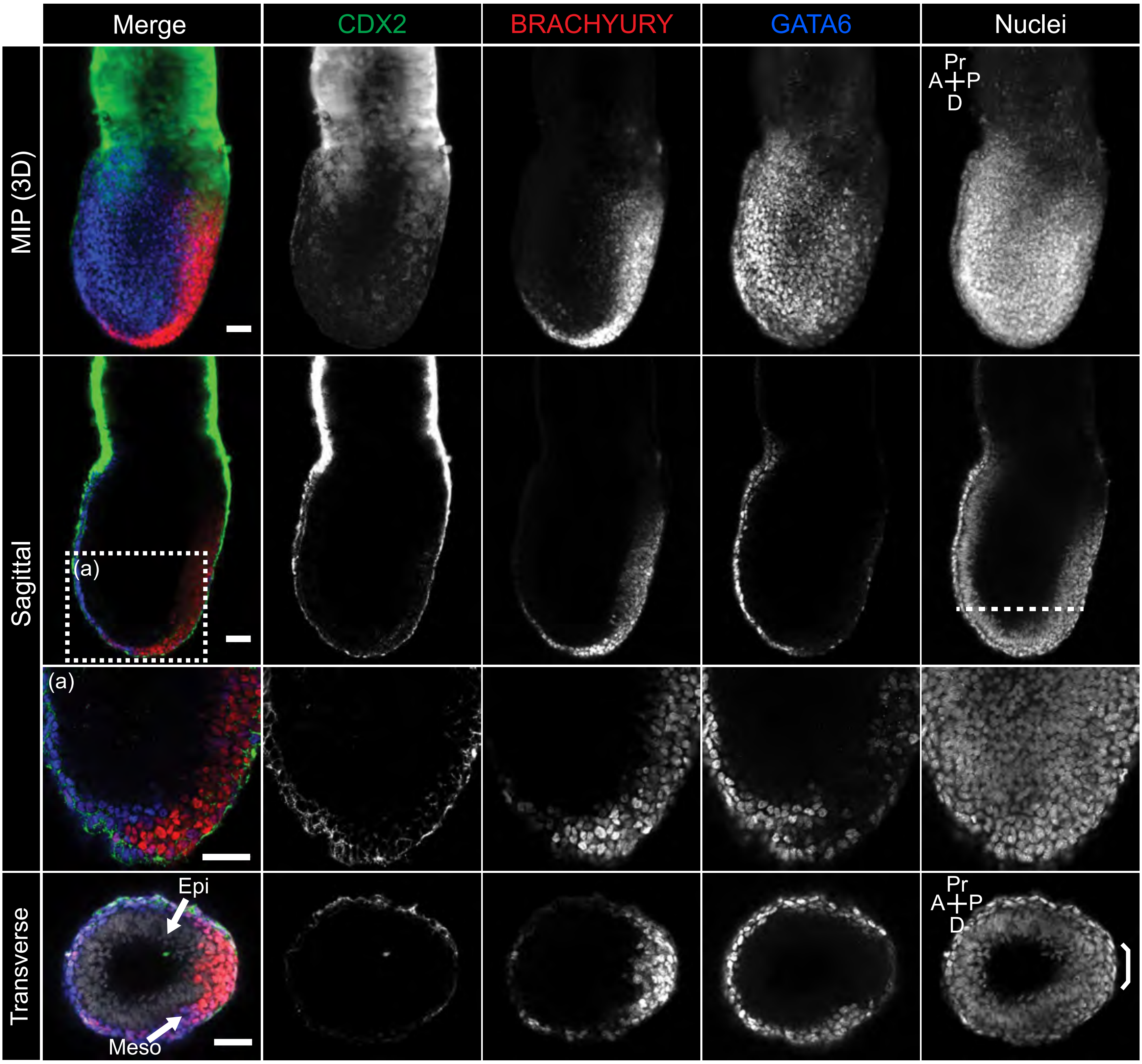

Morgani et al., xii. CDX2, BRACHYURY, GATA6: E7.75

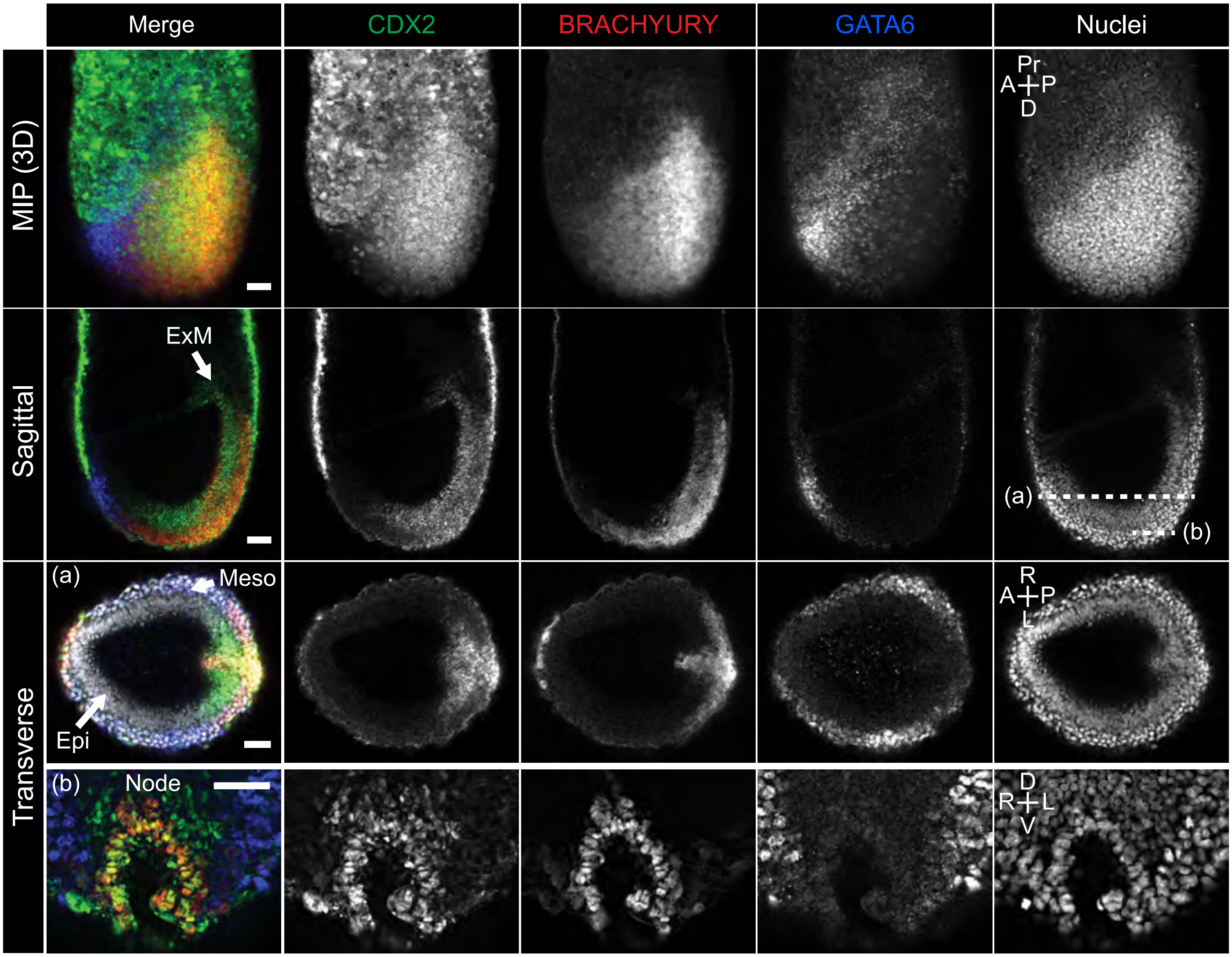

Morgani et al., xii. CDX2, BRACHYURY, GATA6: E7.75

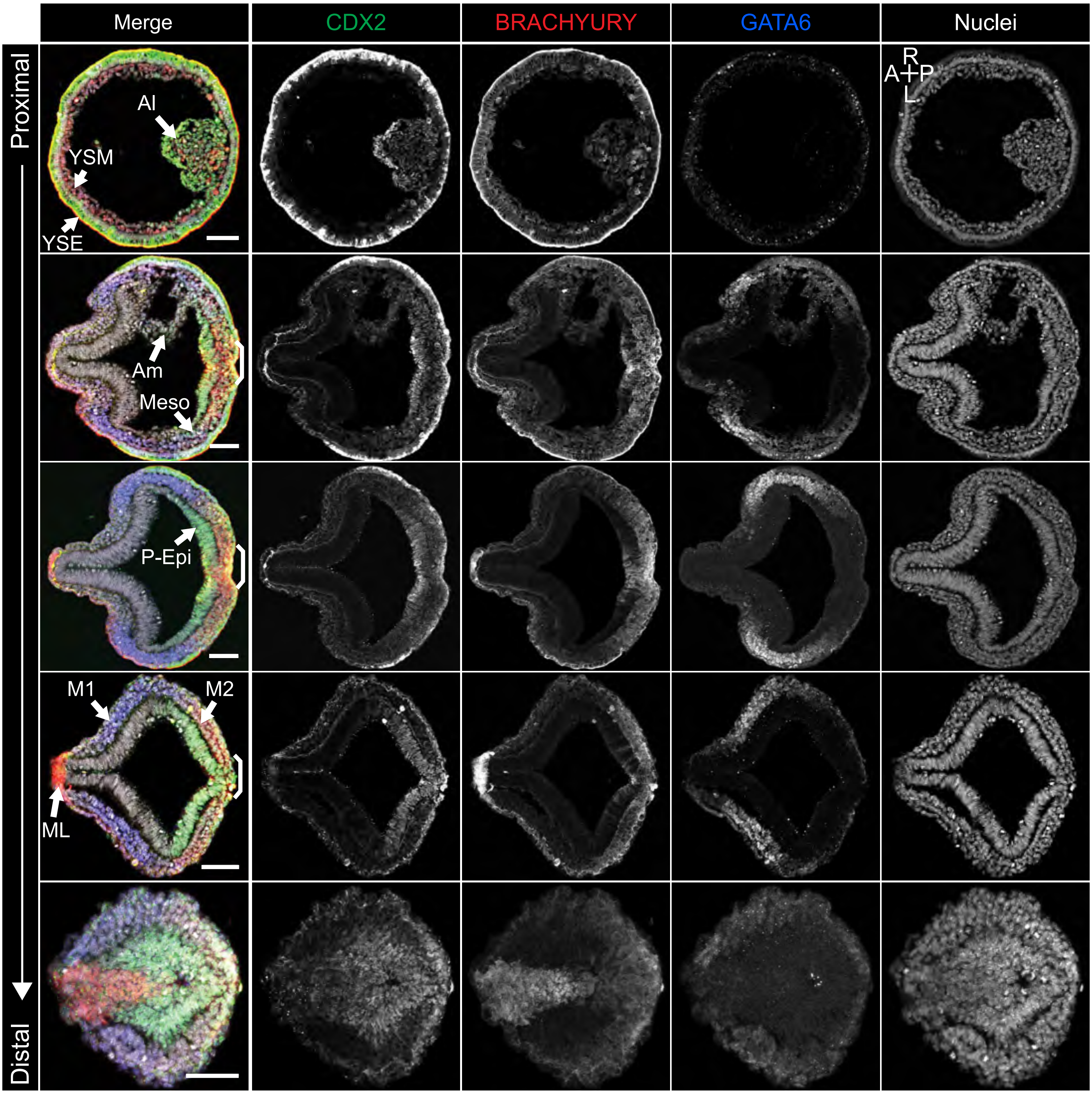

Yellow arrowhead indicates BRACHYURY/GATA6 -expressing cell.
Blue arrowhead: Non-nuclear CDX2 staining in visceral endoderm.

Morgani et al., xiv. CDX2, SOX17, GATA6: E6.5-E6.75

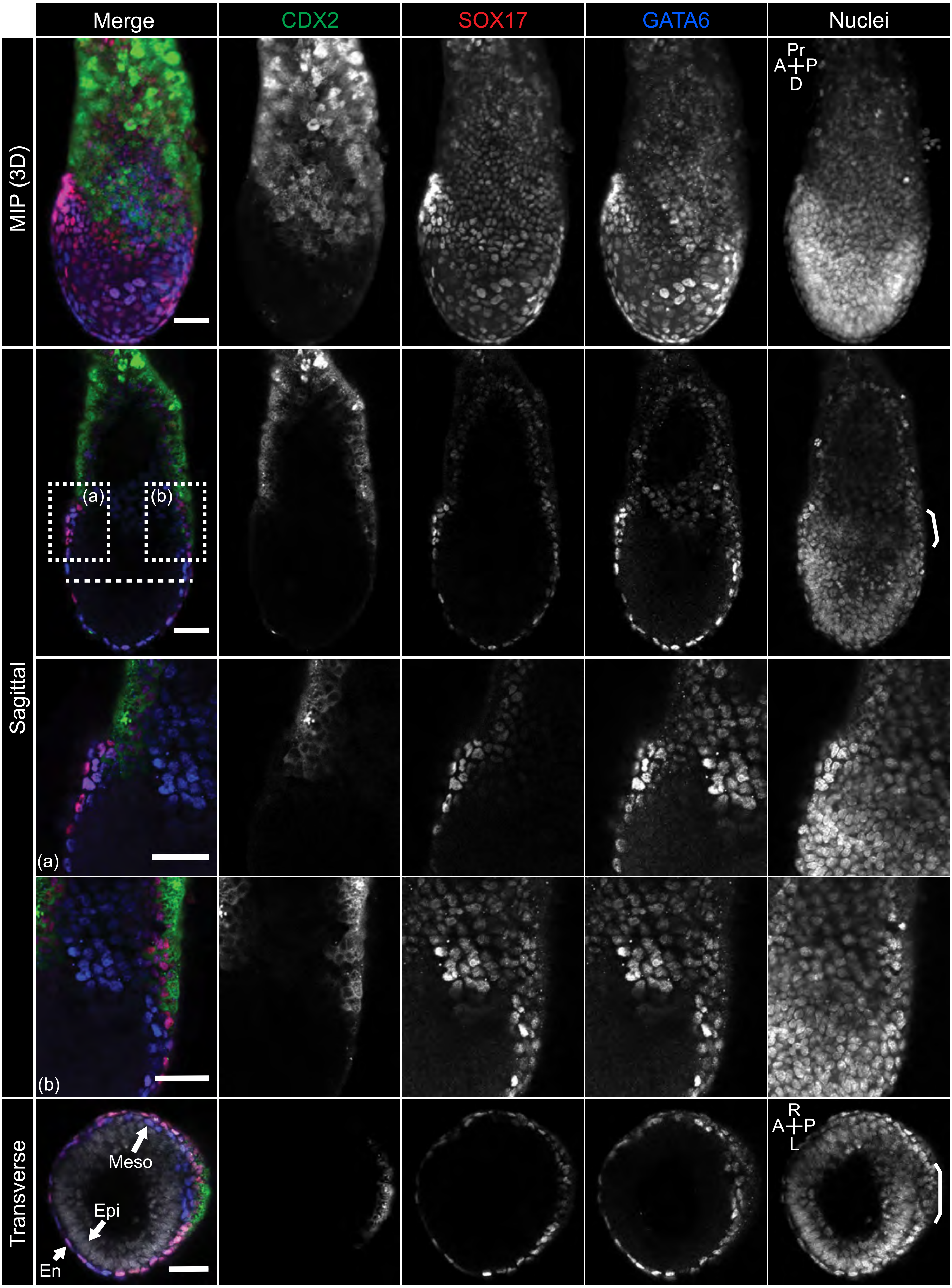

Morgani et al., xv. CDX2, SOX17, GATA6: E6.5-E6.75

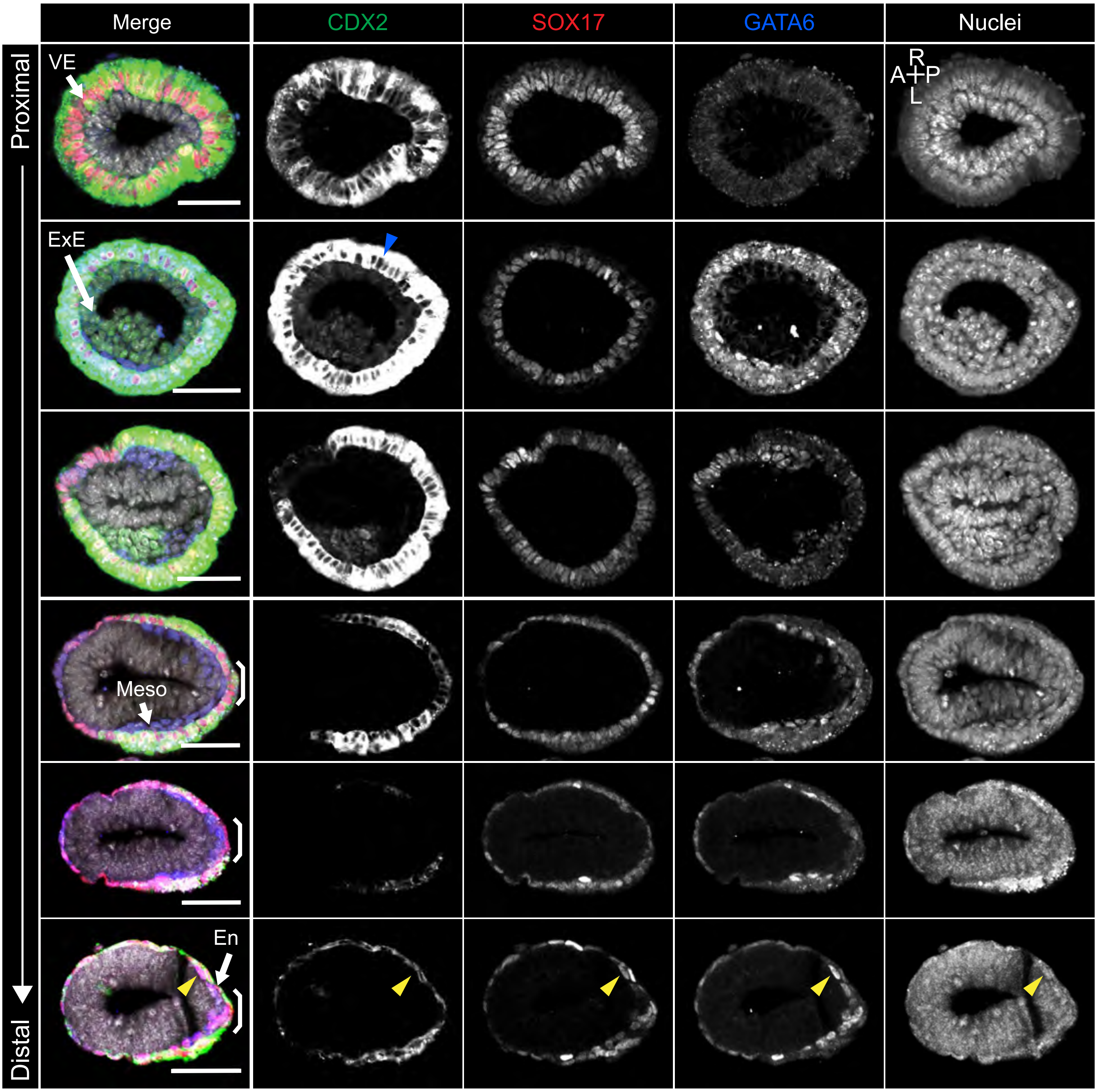

Blue arrowhead: Non-nuclear CDX2 staining in extraembryonic visceral endoderm.
Yellow arrowhead: GATA6/SOX17 -expressing cell.

Morgani et al., xvi. CDX2, SOX17, GATA6: E7.75-E8.0

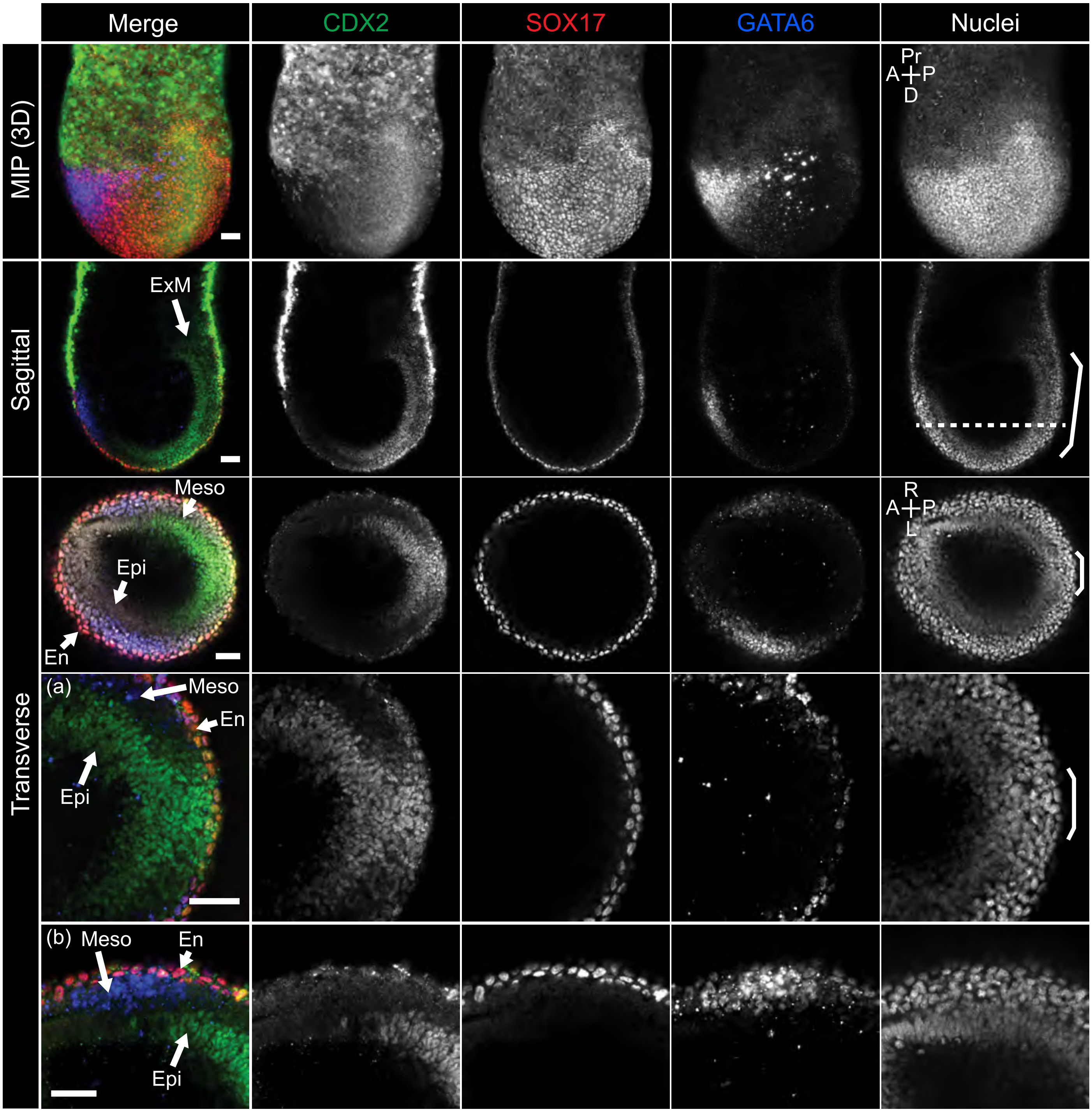

Morgani et al., xvii. CDX2, SOX17, GATA6: E7.75-E8.0

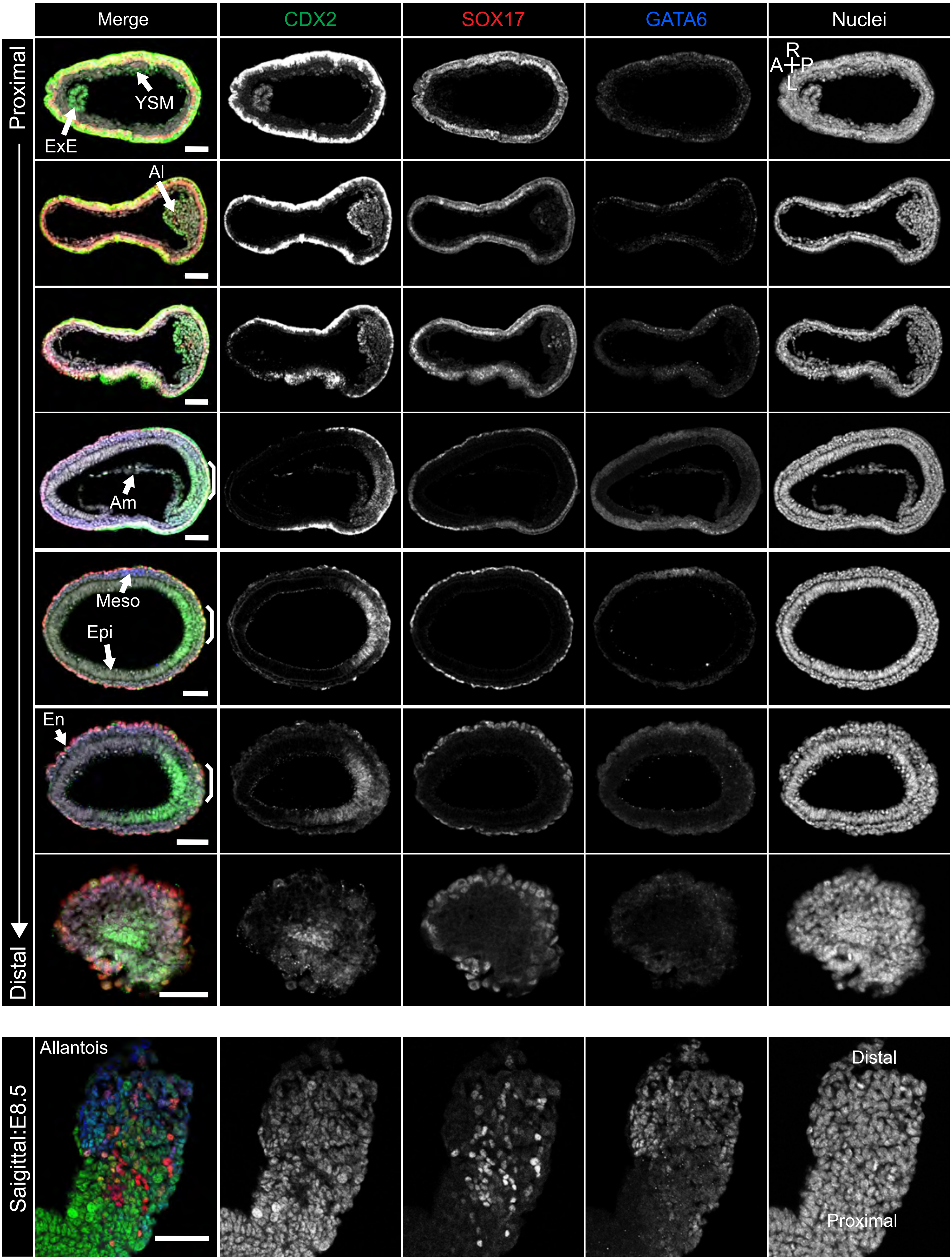

Morgani et al., xviii. FOXA2, BRACHYURY, CDX2: E7.0-E7.25

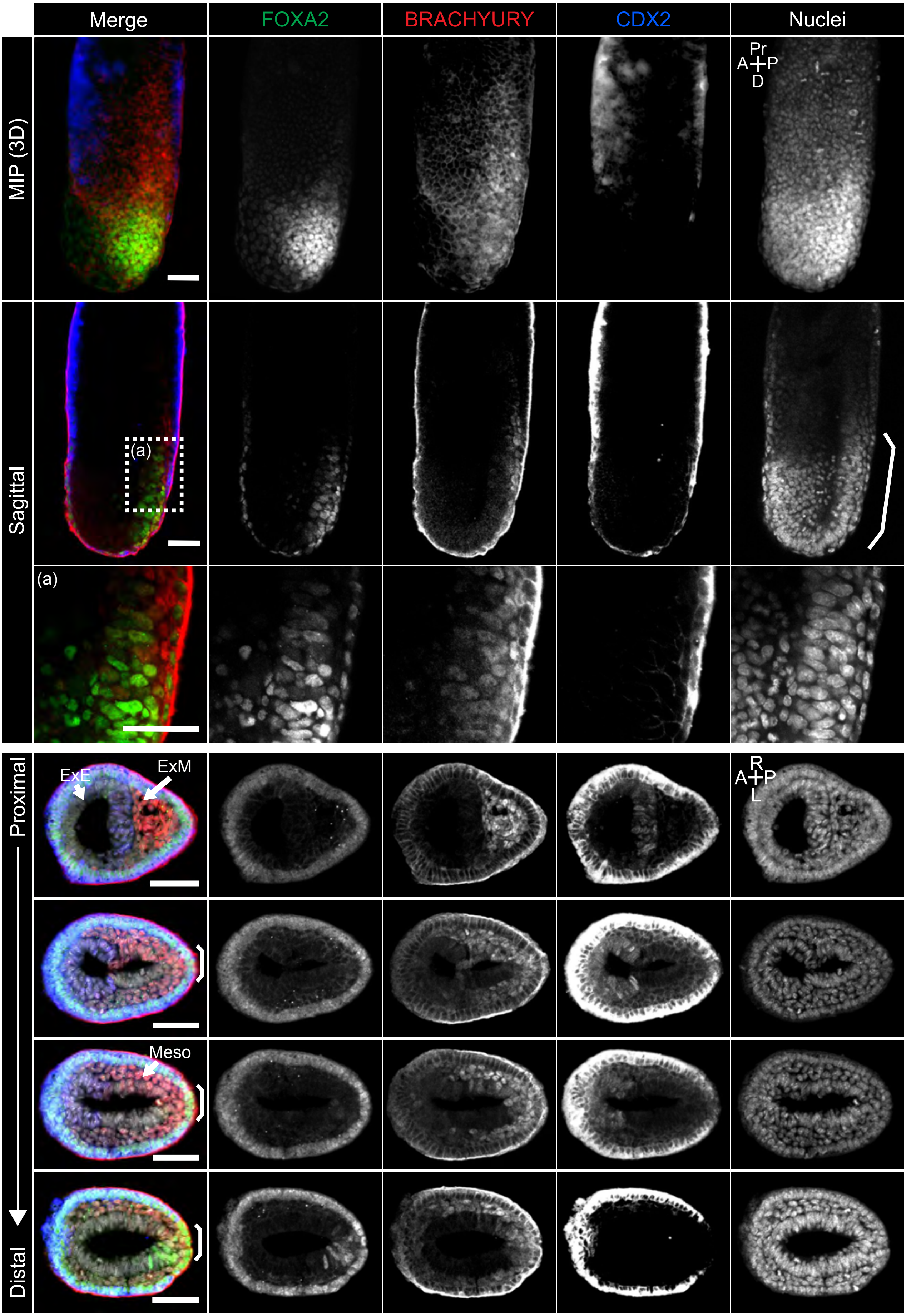

Non-nuclear signal for anti-BRACHYURY/anti-CDX2 antibodies likely represents non-specific staining.

Morgani et al., xix. FOXA2, BRACHYURY, CDX2: E7.5-E7.75

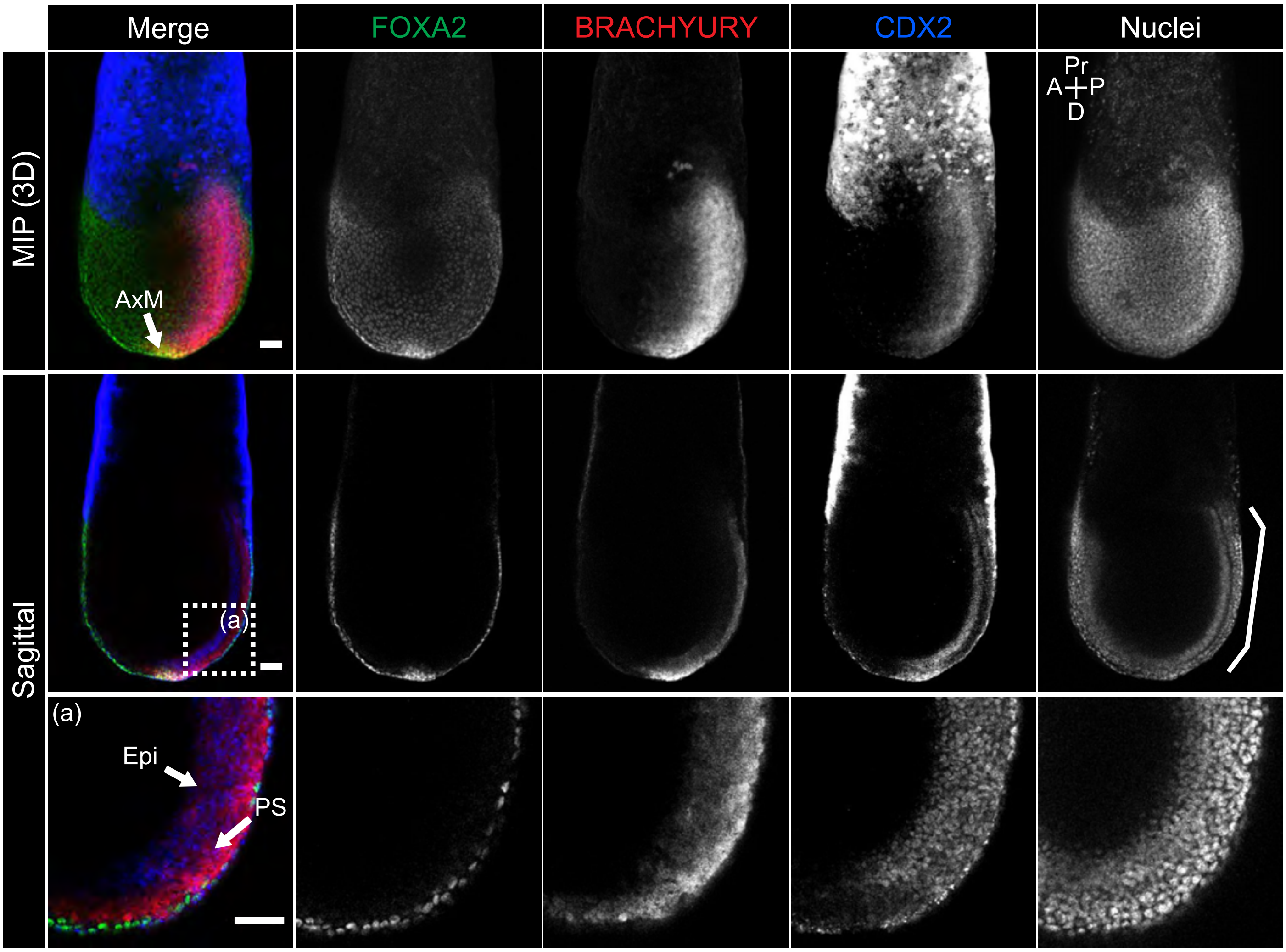

Morgani et al., xx. FOXA2, BRACHYURY, CDX2: E7.5-E7.75

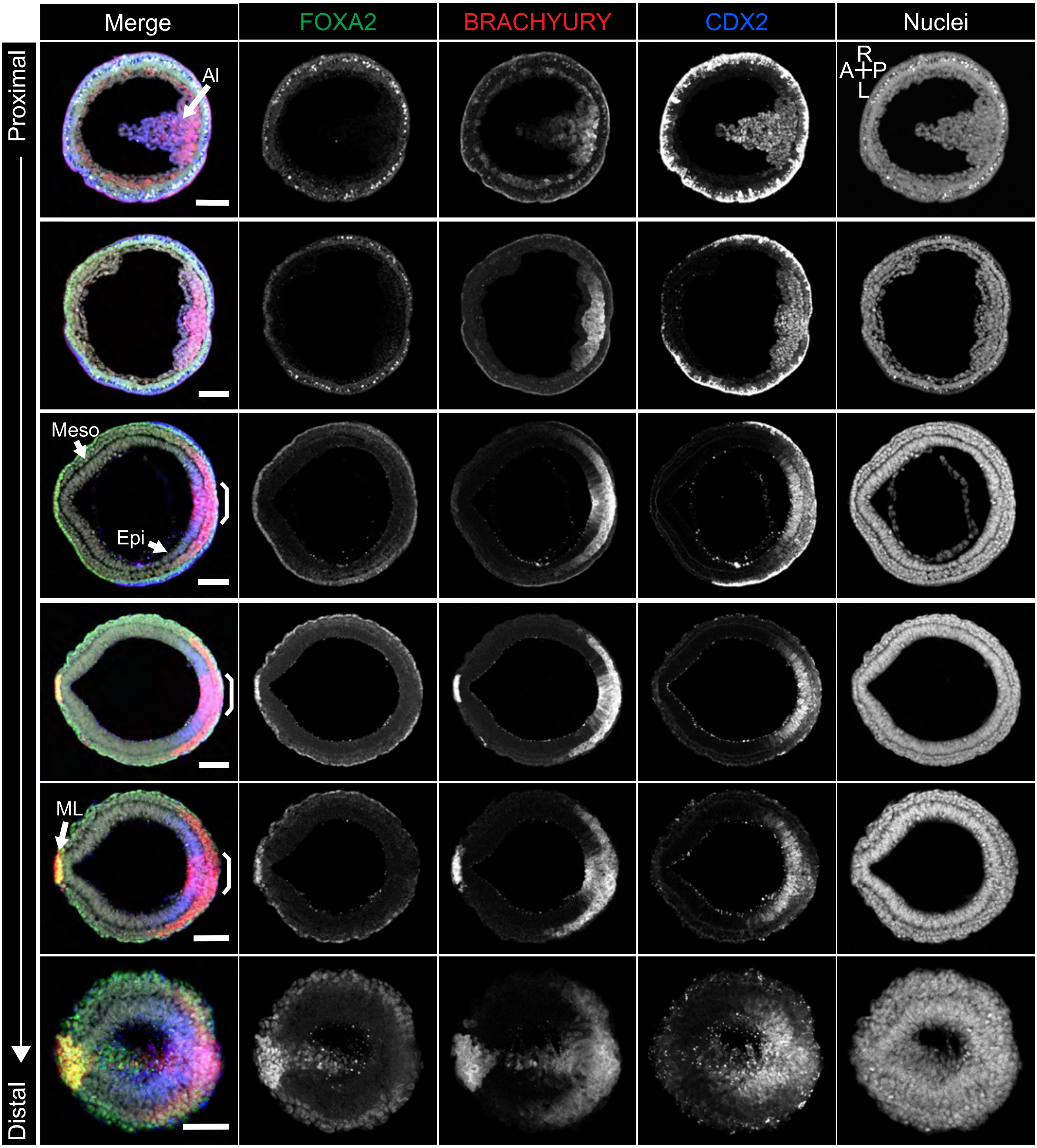

Morgani et al., xxi. FOXA2, SOX17, CDX2: E7.0-E7.25

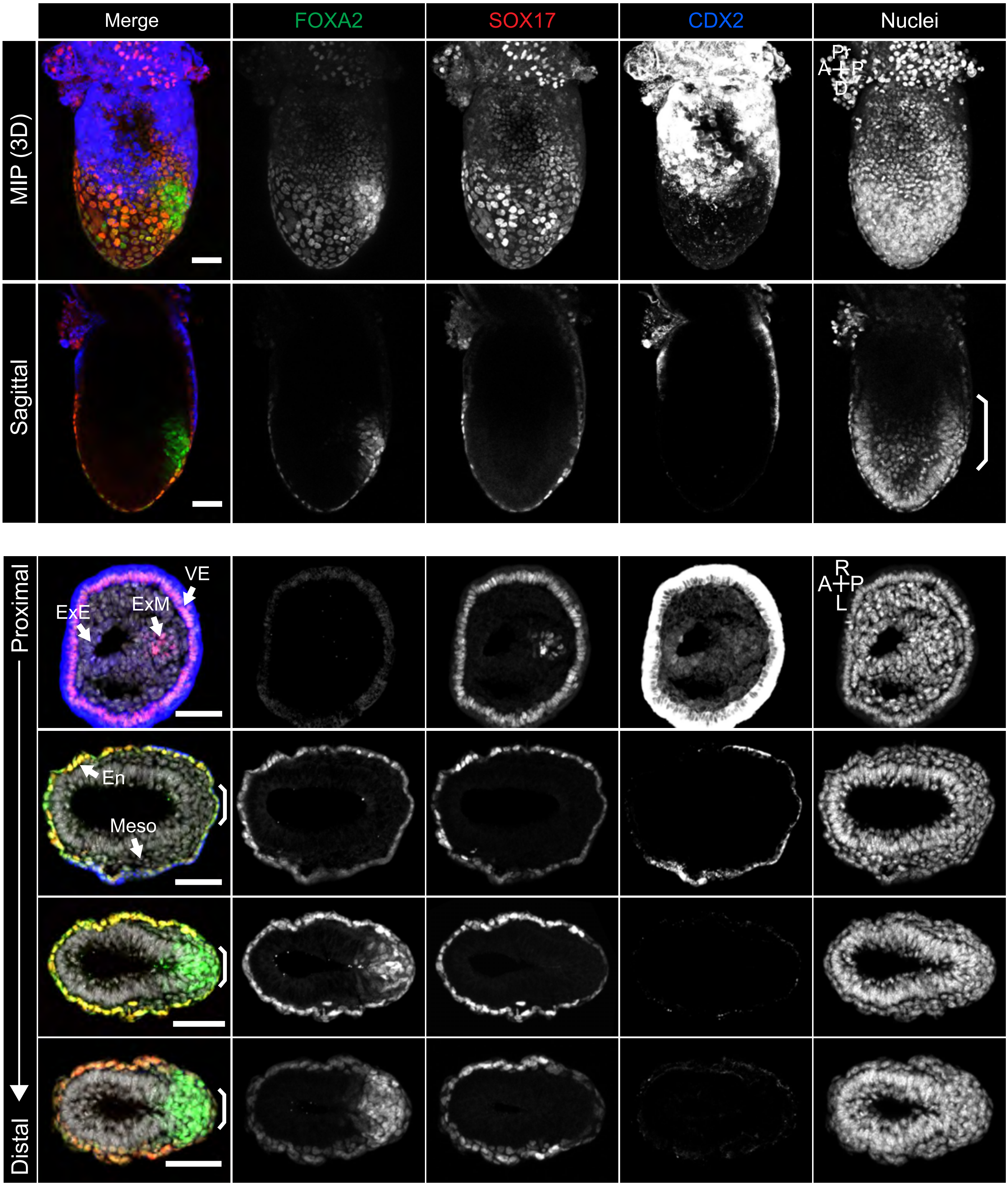

Morgani et al., xxii. FOXA2, SOX17, CDX2: E7.75-E8.0

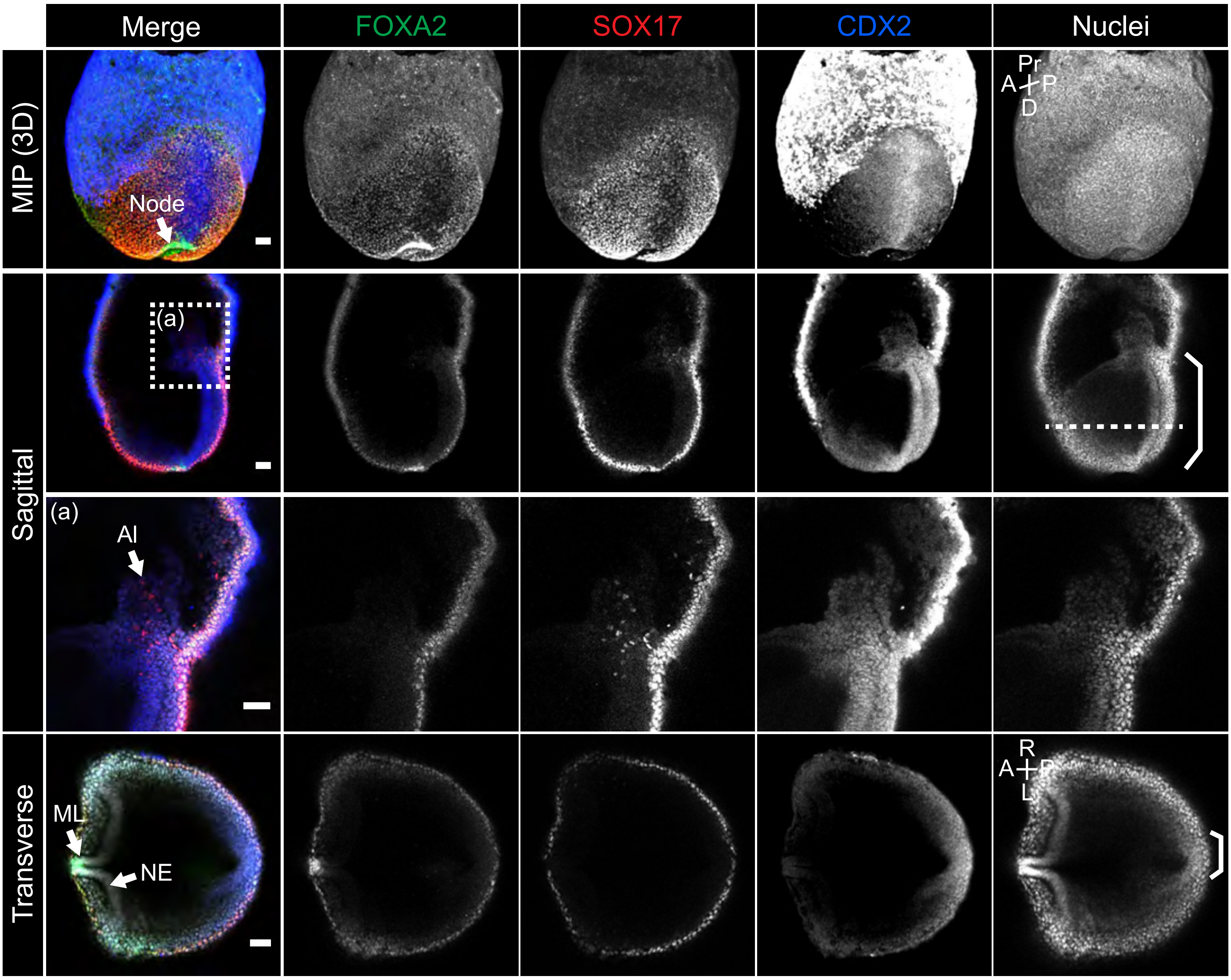

Morgani et al., xxiii. FOXA2, SOX17, CDX2: E7.75-E8.0

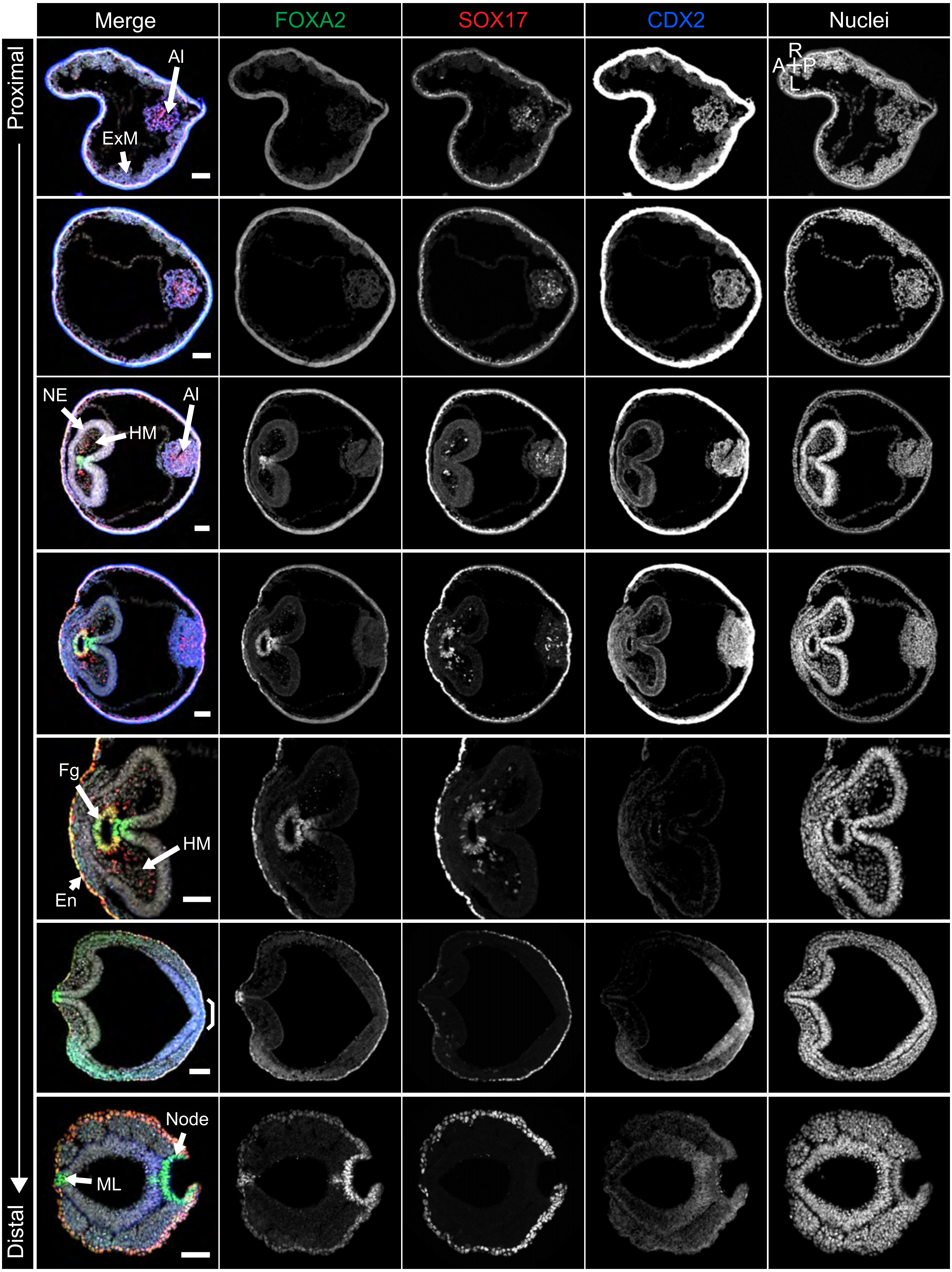

Morgani et al., xxiv. FOXF1, GATA6, CDX2: E6.5-E6.75 and E7.75-E8.0

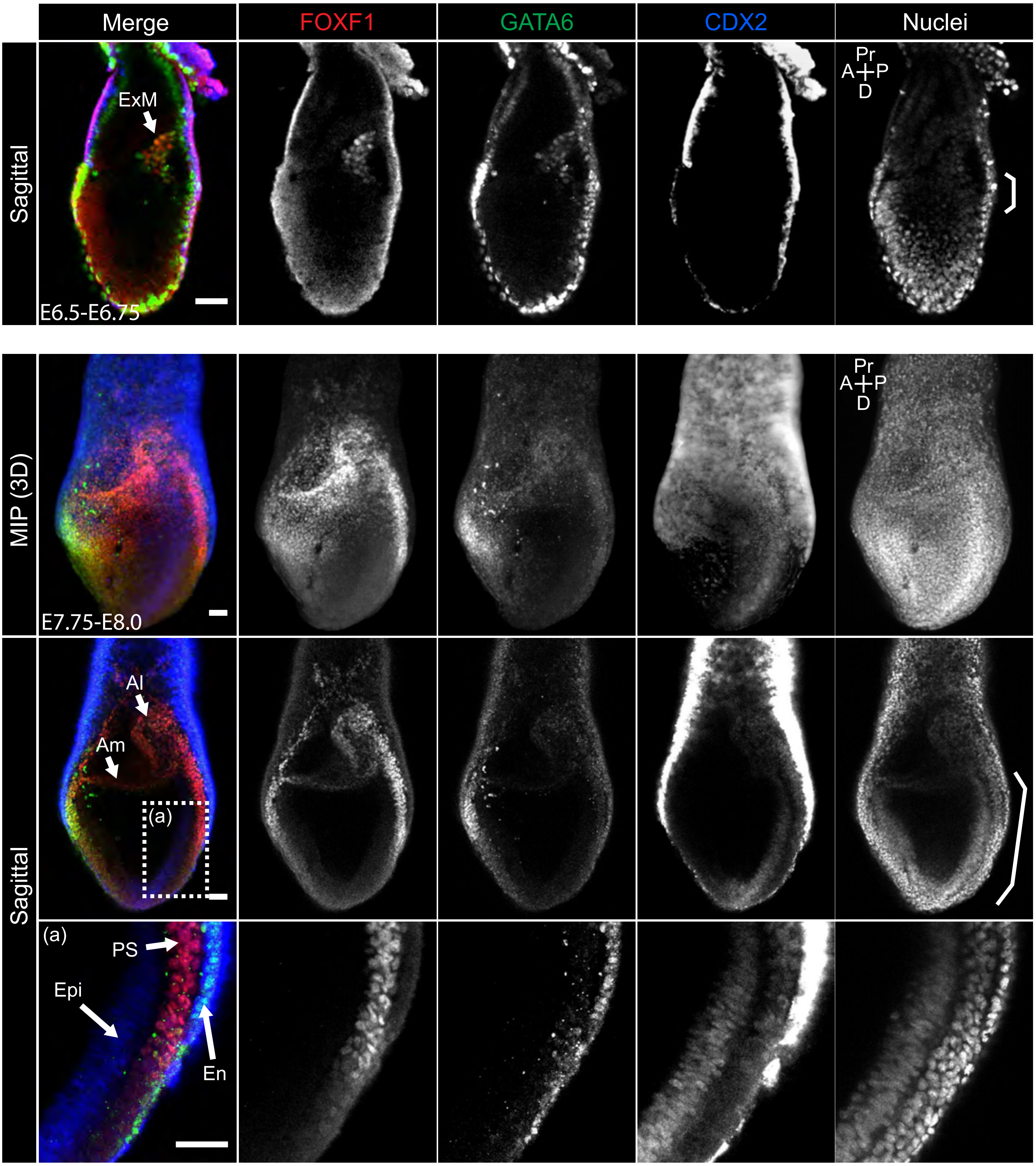

Morgani et al., xxv. FOXF1, GATA6, CDX2: E7.75-E8.0

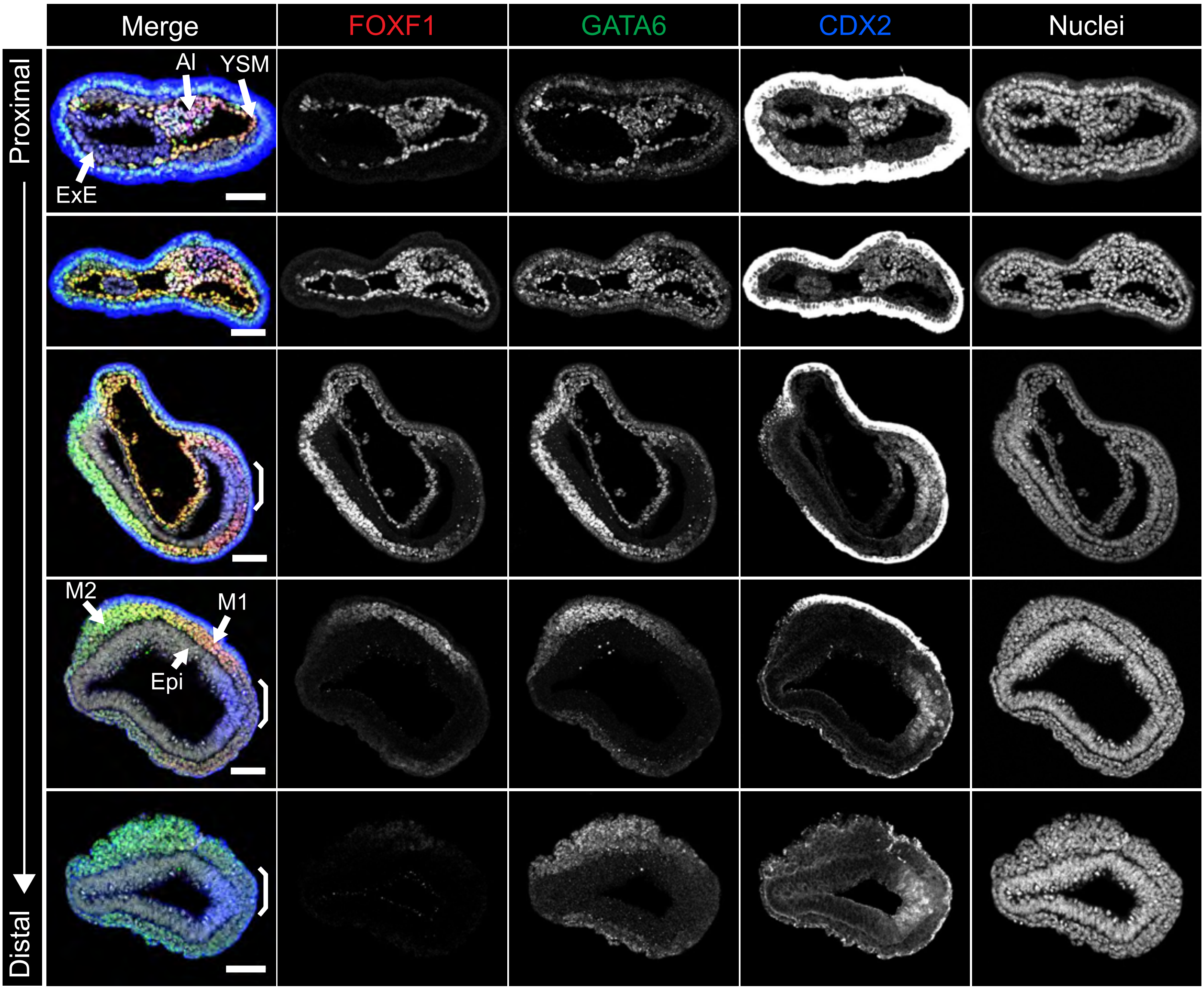

Morgani et al., xxvi. OTX2, SOX2, NANOG: E6.25 and E7.5

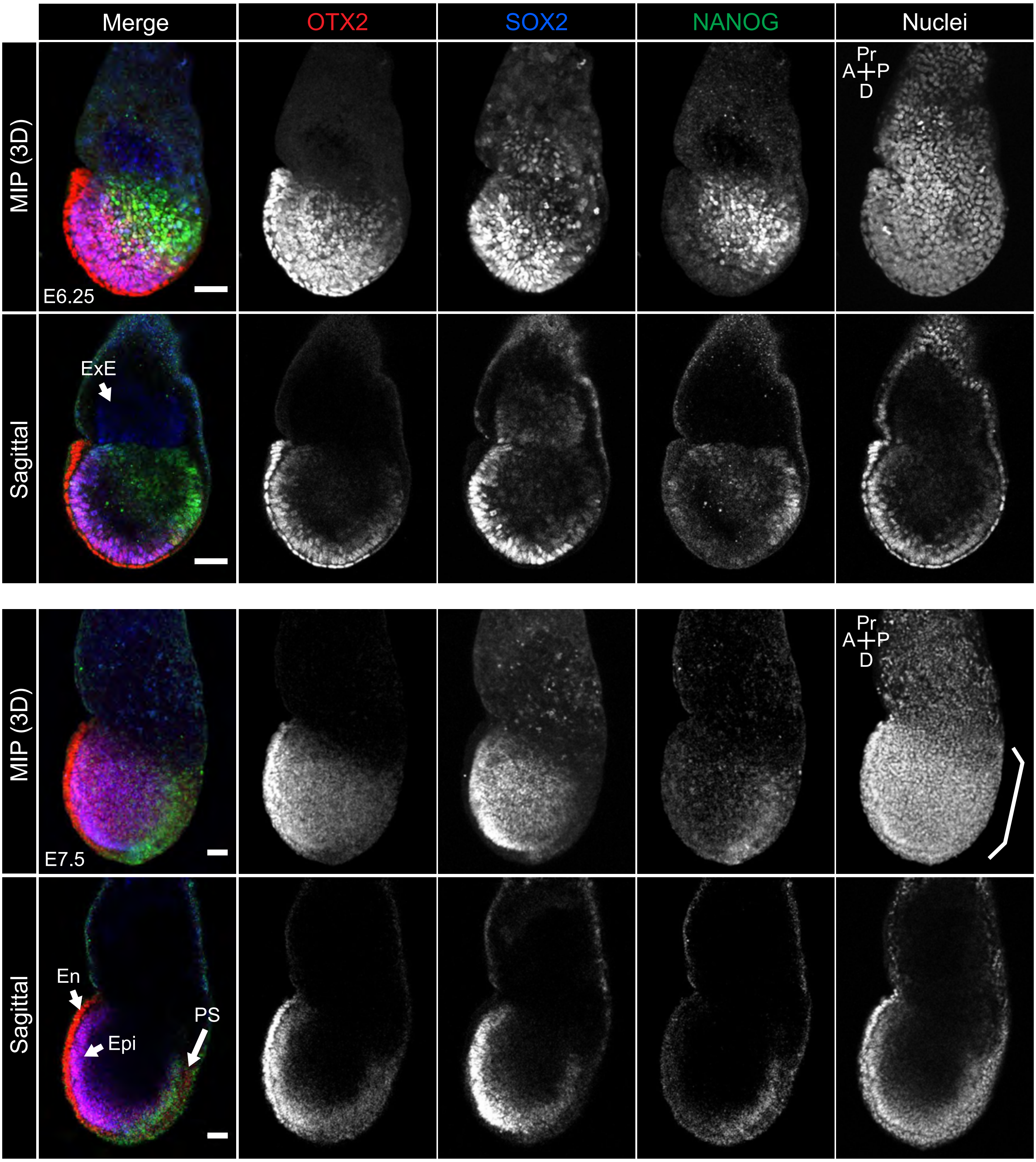

Morgani et al., xxvii. OTX2, SOX2, NANOG: E7.5

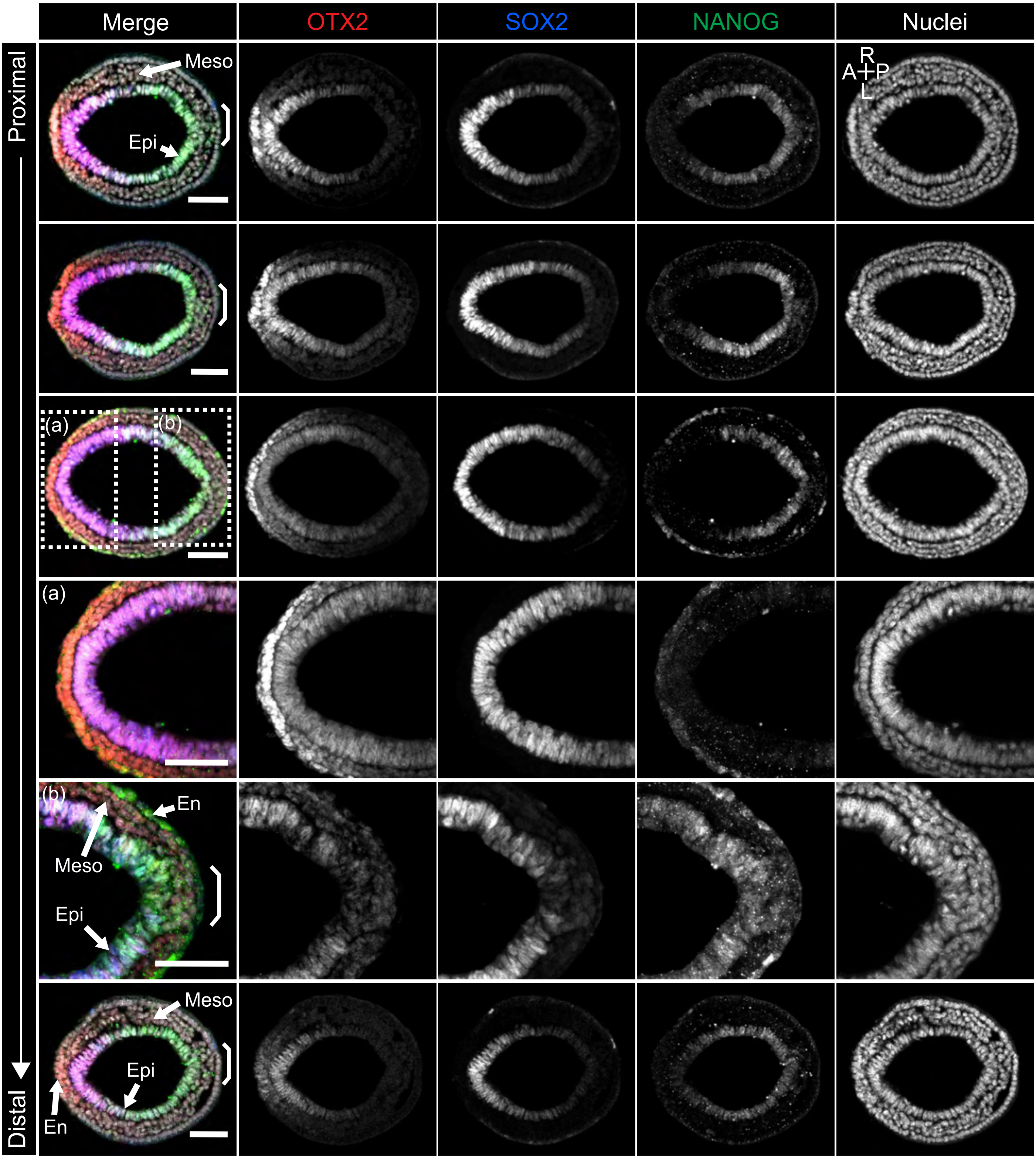

Morgani et al., xxviii. OTX2, SOX2, NANOG: E7.75-E8.0

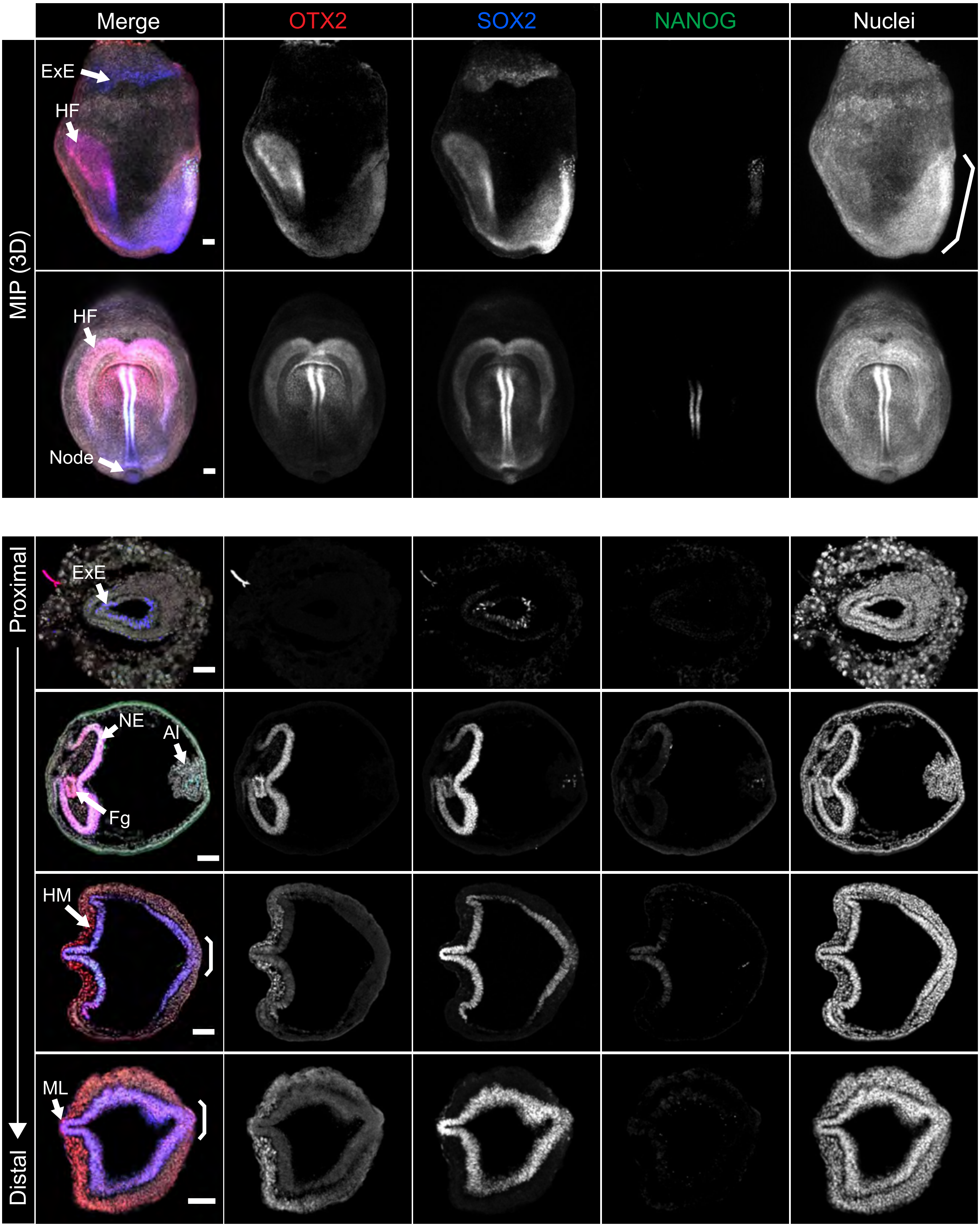

Morgani et al., xxix. pSMAD1/5/8, BRACHYURY, CDH1: E6.5-E6.75

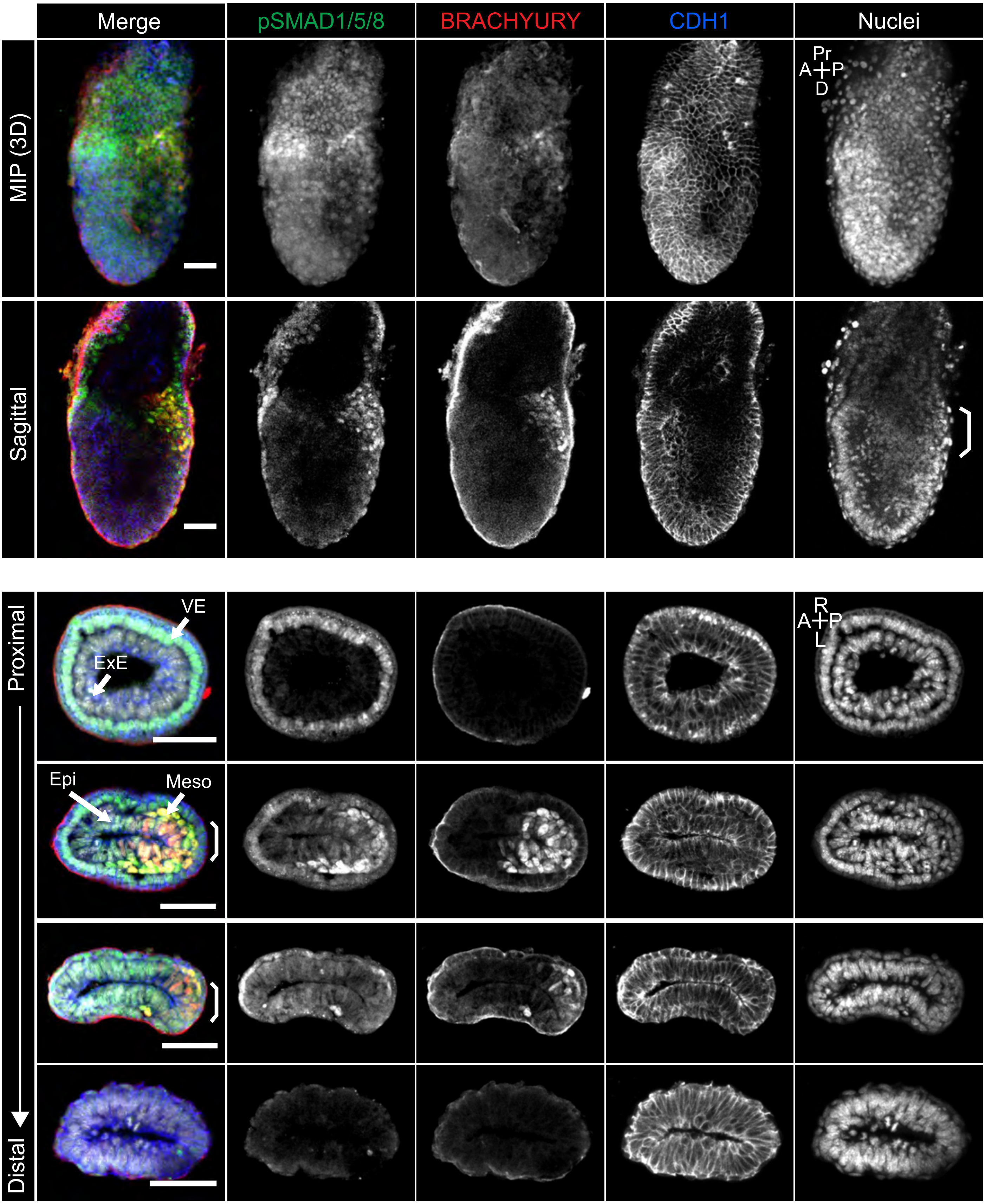

Morgani et al., xxx. pSMAD1/5/8, BRACHYURY, CDH1: E7.0-E7.25

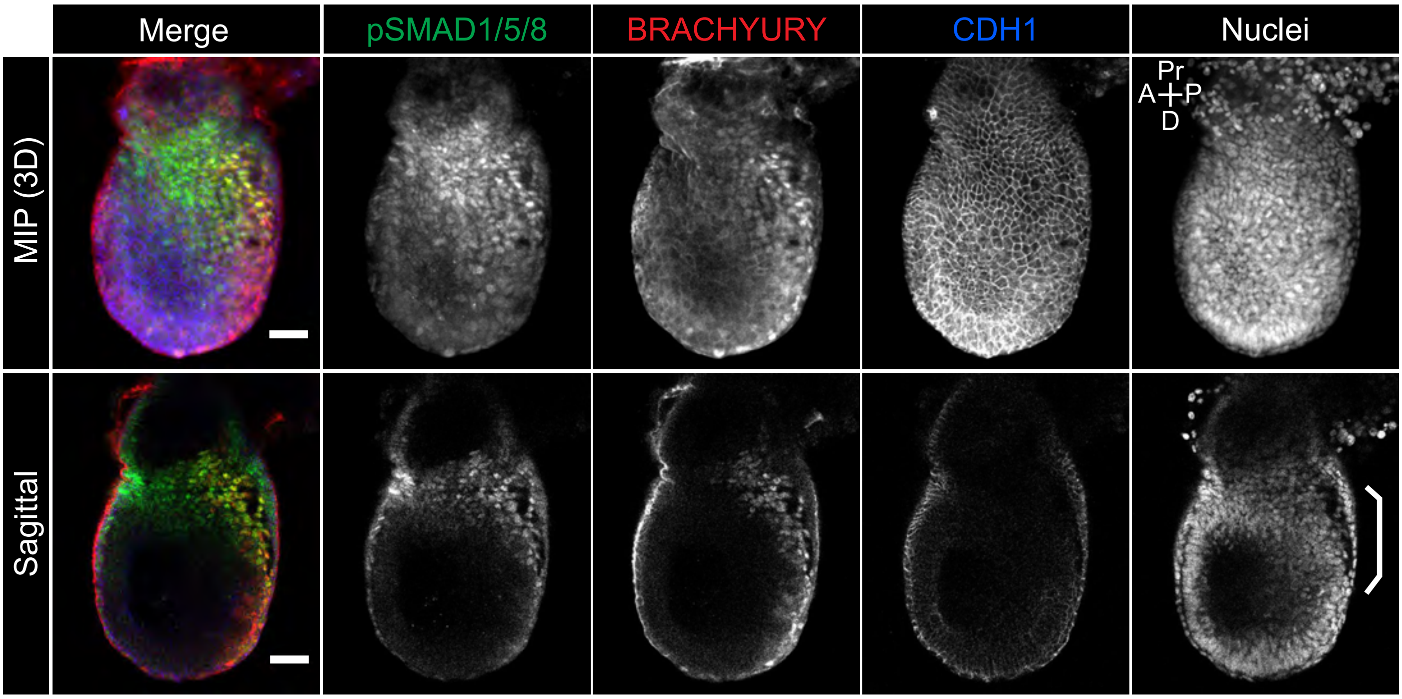

Morgani et al., xxxi. pSMAD1/5/8, BRACHYURY, CDH1: E7.75-E8.0

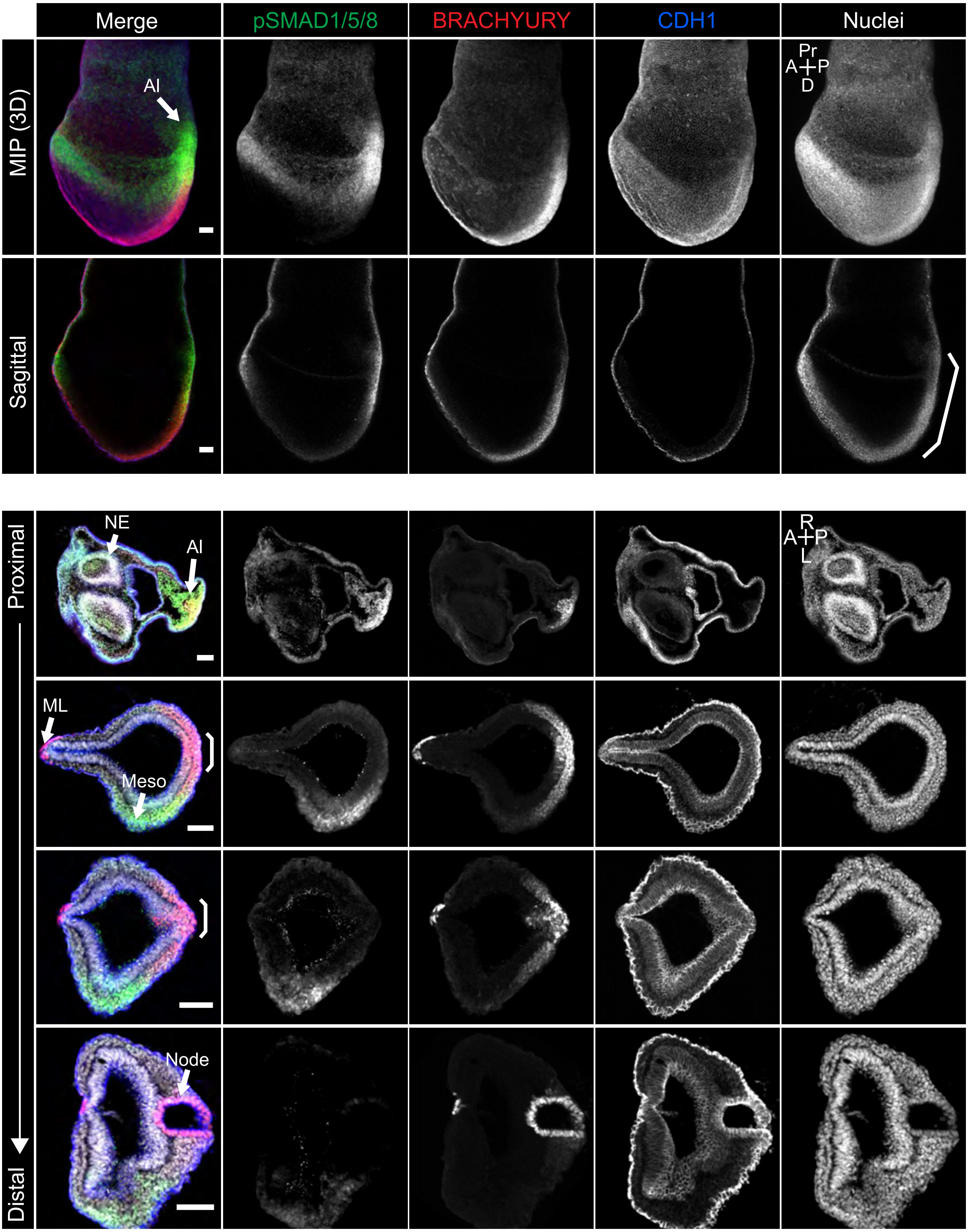

Supplement: Supplementary file 2. — Supplemental file comprising representative images of embryo data used in this study to identify in vivo marker signatures for particular cell states. Embryos were collected at different stages of development throughout gastrulation, from embryonic day (E) 6.25-E8.5 and immunostained for trios of marker combinations. File contains images of wholemount embryos and cryosections acquired by confocal microscopy. The expression patterns of the markers determined using this data are summarized in Supplementary file 1. [file elife-32839-supp2.pdf]
